# Nonheme Iron Catalyst Selectively Activates Oxygen to Hydrogen Peroxide

**DOI:** 10.1021/jacsau.5c00320

**Published:** 2025-06-11

**Authors:** Hsien-Liang Cho, Daoyang Zhang, Alison R. Fout

**Affiliations:** Department of Chemistry, 14736 Texas A&M University, 580 Ross St., College Station, Texas 77843, United States

**Keywords:** secondary coordination sphere, nonheme system, oxygen activation, hydrogen peroxide production, two-proton/two-electron selectivity

## Abstract

Iron complexes are known for their excellent reactivity toward the oxygen reduction reaction (ORR), which proceeds via two possible pathways: a two-electron/two-proton (2e^–^/2H^+^) process to form hydrogen peroxide or a four-electron/four-proton (4e^–^/4H^+^) process to form water. Developing catalysts that enable selective oxygen reduction remains a challenge. Inspired by heme-based systems, we designed two iron complexes incorporating secondary coordination sphere interactions to investigate their influence on the ORR selectivity. The complexes, [Py_2_Py­(afa^Cy^)_2_Fe]­OTf_2_ and [N­(afa^Cy^)_3_Fe]­OTf_2_, were evaluated for their catalytic activity using decamethylferrocene as the reductant, with reaction progress monitored via absorbance spectroscopy. [Py_2_Py­(afa^Cy^)_2_Fe]­OTf_2_ exhibited a selectivity profile comparable to iron porphyrin but with a slower kinetic rate, likely due to the steric hindrance from ligand functionalization. [N­(afa^Cy^)_3_Fe]­OTf_2_ demonstrated exceptional selectivity toward the 2e^–^/2H^+^ pathway, a rare observation for nonheme iron complexes. Kinetic measurements revealed that the catalytic reaction with [N­(afa^Cy^)_3_Fe]­OTf_2_ follows second-order kinetics with a rate constant of 81 mM^–1^ s^–1^. We propose that the rate-determining step involves electron transfer from decamethylferrocene to the hydroperoxo iron­(III) complex, occurring through a stepwise proton transfer/electron transfer (PTET) or electron transfer/proton transfer (ETPT) process, followed by hydrogen peroxide dissociation.

## Graphical abstract

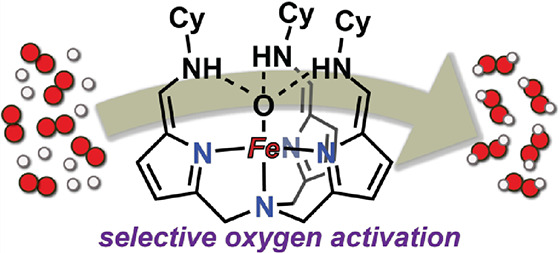

## Introduction

The activation of small molecules using first-row transition metal complexes has been extensively studied over the past few decades.^1^ Among these, metalloporphyrinoid complexes have garnered significant attention due to three unique advantages. First, porphyrinoid ligands provide a stable tetradentate coordination environment capable of chelating many first-row transition metals in their +2 or +3 oxidation states with an additional axial X-type ligand. These geometries allow for an axial vacant site where small molecules can associate with the metal center. Second, metalloporphyrin complexes are chemically stable under a variety of catalytic conditions, including acidic, basic, and aqueous environments. Third, the meso and β positions of the porphyrin framework are highly reactive, enabling functionalization with diverse substituents to modulate the electronic and steric properties of the metal center.^2,3^

Over the past few decades, novel porphyrinoid derivatives have been developed for the activation of small molecules relevant to energy and environmental applications, such as O_2_,^4–6^ CO_2_,^7,8^ H_2_,^9,10^ and some oxyanions.^11^ In particular, O_2_ activation is of considerable interest due to its role in the oxygen reduction reaction (ORR), which serves as the cathodic reaction in hydrogen fuel cells. The 4e^–^/4H^+^ pathway generates water as a clean byproduct while providing 1.23 V of electrical energy.^12^ Alternatively, the 2e^–^/2H^+^ pathway is a promising route for hydrogen peroxide production.^13,14^

Despite the advantageous properties of metalloporphyrinoid complexes in ORRs, it is rare for a monomeric porphyrinoid complex to exhibit exclusive selectivity toward either 4e^–^/4H^+^ or 2e^–^/2H^+^ pathway.^15^ To improve selectivity, modification of the porphyrinoid complexes was explored. These include the incorporation of an additional metal site at a proper distance for facial or dinuclear enhancement,^16–18^ which facilitated selective 4e^–^/4H^+^ chemistry, as well as disrupting the aromaticity of the porphyrin to alter the electronic structure of the complexes.^19^ Additionally, a pretemplated metal–metal distance greater than 10 Å can favor the 2e^–^/2H^+^ chemistry.^13,14^

Beyond electronic and coordination environment modifications, the second coordination sphere plays a crucial role in influencing ORR reactivities and selectivity.^20^ Cytochrome c oxidase, for example, catalyzes the four-electron reduction of oxygen to water at a binuclear active site, composed of a high-spin heme iron complex and a copper moiety.^21^ In well-studied mechanisms, a metal (hydro)­peroxo intermediate is stabilized via the hydrogen bonding network involving tyrosine residues, facilitating the coupling of proton transfer and electron flow.^22,23^ This network drives proton pumping and contributes to the proton gradient used in ATP synthesis.^24^ Inspired by biological systems, biomimetic complexes with secondary coordination spheres have been shown to enhance selectivity in the ORR.^12,20,25,26^

Despite these advances, the practical application of highly selective catalyst designs is hindered by challenges in the synthesis. Such syntheses may involve multiple-step syntheses (sometimes exceeding 20 steps), extensive chromatographic purifications,^27^ and even precious metal-assisted coupling reactions, where the precious metals can be directly incorporated into the structural assembly.^16,28^ These synthetic challenges present barriers to systematic mechanistic studies and broader implementations.

While extensive efforts have been conducted on Fe heme-based ORR catalysts, examples of nonheme iron complexes remain surprisingly underexplored (Scheme 1A), despite their relevance to dioxygen activation in catechol dioxygenases^29^ and Rieske dioxygenases.^30,31^ Examples of nonheme iron ORR catalysts include the dinuclear iron­(II) thiolate complex reported by Duboc and co-workers, which demonstrated a tunable selectivity between H_2_O_2_ or H_2_O under chemical or electrochemical conditions, respectively.^32^ Nam and co-workers reported a selective iron­(III) catalyst based on a tetraamido macrocyclic ligand (TAML), [(TAML)­Fe^III^]^−^, that achieves the four-electron reduction of O_2_ via an iron­(V)-oxo intermediate.^33^ Machan and co-workers used [N_3_O]^−^ ligands that bound in either a tripodal or square planar fashion to an iron­(III) catalyst. Both iron complexes undergo a [2 + 2] reaction mechanism, where H_2_O_2_ generated during the catalysis was further reduced by 2e^–^/2H^+^ to give 2 equiv of H_2_O.^34,35^ Paria and co-workers demonstrated that an oxime proton binding site in the secondary coordination sphere accelerates the ORR via an intramolecular proton exchange mechanism, one of the first nonheme examples in which the secondary coordination sphere influences ORR catalysis.^36^ To the best of our knowledge, no other nonheme iron ORR catalyst has been reported to demonstrate any influence of the secondary coordination sphere effects on reactivity.

**Scheme 1:**
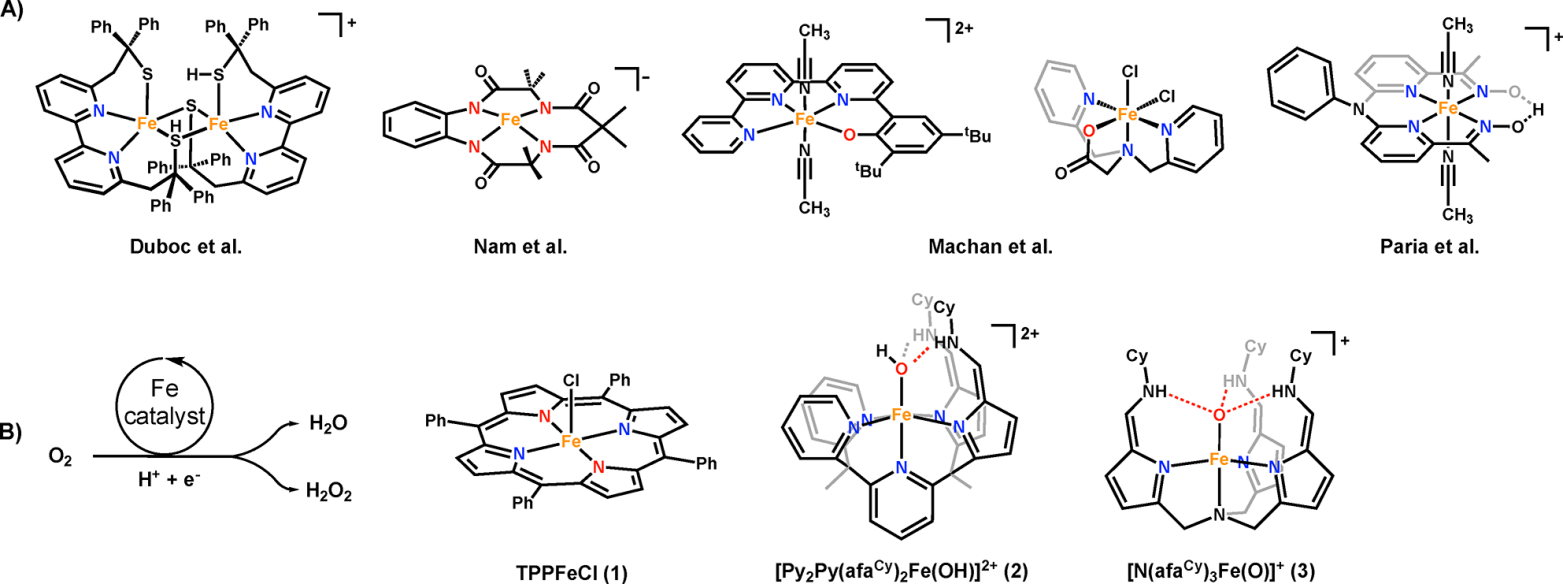
(A) Reported Nonheme Iron-Based Catalysts for Oxygen Reduction Reaction; (B) Iron ORR Catalysts Used in This Study

To gain deeper insights into ORR reactivity in nonheme systems, we selected our recently reported iron complexes for ORR investigation for two main reasons. First, these complexes feature a ligand platform that incorporates in the secondary coordination sphere either hydrogen bonding donors or acceptors through tautomerization.^37^ Second, the synthesis and characterization of the iron complexes are relatively straightforward compared to the porphyrin derivatives, facilitating mechanistic studies.^37–39^ Herein, we compare the ORR reactivity and selectivity between three iron­(III) complexes: (1) a classical porphyrin iron complex (TPPFeCl, **1**, TPP = tetraphenylporphyrin); (2) a tetrapodal iron complex ([Py_2_Py­(afa^Cy^)_2_FeOH]^2+^, **2**, Py_2_Py­(afa^Cy^)_2_ = 2,2′,2′-methyl-bis-pyridyl-6-(2,2′,2′-methylbis-5–5-cyclohexyl-amineazafulvene)-pyridine);^40^ and (3) a tripodal iron-oxo complex ([N­(afa^Cy^)_3_FeO]^+^, **3**, N­(afa^Cy^)_3_ = tris­(5-cyclohexyl-amineazafulvene-2-methyl)­amine),^38^ under both electrocatalytic and electrochemical conditions (Scheme 1B). Additionally, we examined the initial rates of complex **3** using time-resolved UV–vis spectroscopy to monitor the formation of decamethylferrocenium chloride (Fc*Cl) under various conditions. By probing reaction kinetic parameters, mechanistic insights were gleaned in this nonheme system featuring a secondary coordination sphere and its influence on ORR reactivity.

## Results and Discussion

### Synthesis of Iron Precatalysts

The iron precatalysts were synthesized by following previously established procedures. TPPFeCl was prepared via metalation of free base tetraphenylporphyrin with an iron­(II) chloride source, then oxidized by air.^6^ The synthesis of the tetrapodal complex, **2**, was achieved by the addition of potassium bromate to [Py_2_Py­(afa^Cy^)_2_Fe]­OTf_2_, resulting in a color change from yellow to dark brown over the course of 1 h. Filtration and removal of the solvent yielded the desired product **2** in good yield. To prevent proton demetalation of [N­(afa^Cy^)_3_Fe]­OTf_2_, triethylamine was added before exposure to oxygen.^38^ The immediate color change from yellow to dark brown also confirmed the formation of targeted tripodal complex **3**. All paramagnetic nonheme iron­(III) complexes were characterized using ^1^H NMR spectroscopy, with spectral data consistent with previous reports.^38,40^

### Electrochemical Oxygen Reduction

To evaluate the electrical catalytic activity of the iron complexes, homogeneous cyclic voltammetry experiments (CVs) were conducted under four different conditions (Figure 1a–c): (1) Red trace: Under N_2_, with catalyst, but in the absence of a proton source; (2) Blue trace: Under O_2_, with catalyst, but in the absence of a proton source; (3) Green trace; Under N_2_, with trifluoroacetic acid (TFA) as a proton source, and catalyst; and (4) Purple trace: Under O_2_, TFA is used as proton source and catalyst. As observed by the red trace under N_2_, there was no catalytic response, as depicted in the noncatalytic cyclic voltammograms for **2** and **3** (Figure S56). The redox event at *E*_1/2_ value of–0.6 V vs ferrocene for **2** and *E*_1/2_ value of −0.3 V for **3** can be attributed to the Fe^III/II^ couple. In the presence of O_2_ (blue trace), a noncatalytic reversible superoxide formation was detected at the glassy carbon working electrode with an *E*_1/2_ value of −1.2 V vs FcH^+^/FcH. Upon addition of TFA as proton source under an N_2_ atmosphere (green trace), catalytic waves with *E*_onset_ potentials −1.2 V for **2** and −1.1 V for **3**, that are more negative than −1 V vs FcH^+^/FcH, were observed, which we ascribe to the hydrogen evolution reaction (HER) catalyzed by the iron complexes. Finally, under an O_2_ atmosphere with TFA present (purple trace), an appreciable current response was observed at an onset potential far more positive than that for the HER, consistent with electrochemical oxygen reduction. Among the three catalysts, **1** exhibited the most positive *E*_onset_ at ca. −0.1 V, whereas the *E*_onset_ of **2** is at ca. −0.5 V, and the *E*_onset_ of **3** is at ca. −0.3 V vs FcH^+^/FcH (Figure 1f). Interestingly, for **3**, an *E*_1/2_ value of ca. −0.6 V can be observed for the Fe_II/III_ (Figure S56). At the same time, the *E*_onset_ for ORR was measured as ca. −0.3 V, suggesting that the precatalyst was reduced to Fe^II^, which may undergo a proton-coupled electron transfer (PCET) mechanism to form the Fe^II^ active species. The hypothesized PCET mechanism was also established by mixing decamethylferrocene (Fc*) as a chemical reductant with complex **3**. Before adding external acid, Fc*^+^ cannot be observed in the absorption spectra, suggesting that the Fc* is not strong enough reductant to reduce complex **3**; yet with the addition of acid, the successful reduction of Fe^III^ can be achieved, accompanied by the formation of Fc*^+^ (Figure S54). With this hypothesis mechanism proposal, a deeper mechanistic study using the chemical reduction method was conducted.

**Figure 1:**
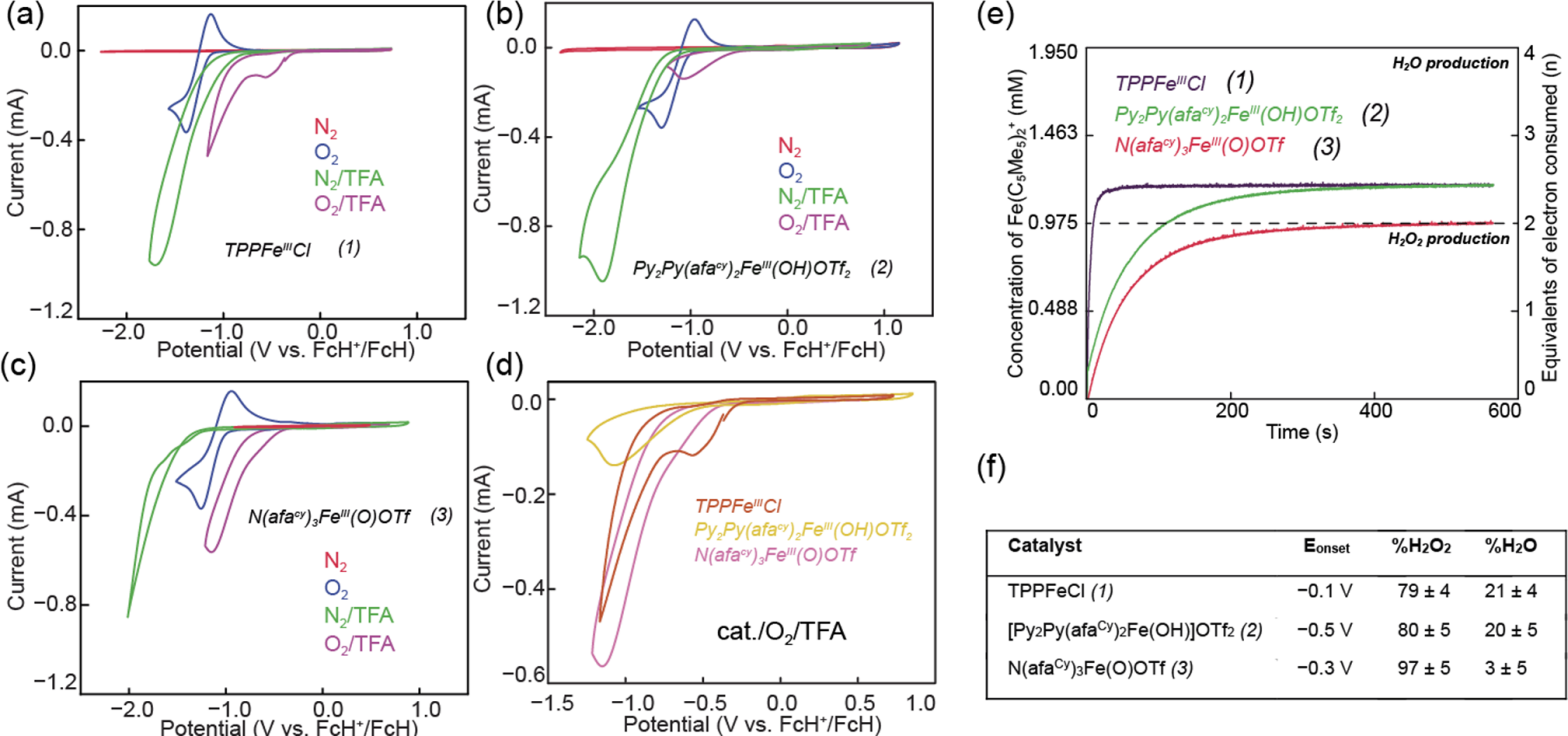
CVs of Fe catalysts under homogeneous conditions. For (a–c). Red: 0.1 mM iron catalyst under a N_2_ atmosphere. Blue: 0.1 mM iron catalyst under the O_2_ atmosphere. Green: 0.1 mM iron catalyst under N_2_ atmosphere with 100 mM TFA. Purple: 0.1 mM iron catalyst under O_2_ atmosphere with 100 mM TFA. For (d), the CVs of catalytic oxygen activation for three iron catalysts was stacked; the condition is identical with the purple traces from previous three CVs. In all cases, dry acetonitrile with 100 mM TBAPF_6_ was used. Scan rate: 100 mV s^–1^, scan direction: reduction first, FcH^+^/FcH was used as reference. (e) Chemical reduction ORR catalysis by iron complexes that are monitored by decamethylferrocenium formation (λ_max_ = 780 nm, ε = 524.8 M^–1^ cm^–1^ (Figure S50) with initial concentrations of 0.48 mM O_2_, 44 mM HCl, 10 mM decamethylferrocene, and 250 μM catalysts. The concentration of decamethylferrocenium formation was plotted as a function of time. The number of electrons consumed and selectivity were calculated based on the concentration of decamethylferrocenium formation during the reduction. (f) Table of parameters for the iron complexes of hydrogen peroxide and water production evaluated by chemical reduction method, and onset potentials measurement by CV.

### Chemical ORR Studies

To further investigate the ORR behavior of these iron complexes, chemical reduction experiments were targeted using a mixed solvent system of air-saturated DMF and N_2_-saturated saline in the presence of excess decamethylferrocene (Fc*) as the reductant and HCl as the proton source. The reactions were monitored by UV–vis spectroscopy, following the method originally developed by Fukuzumi and Guilard,^41^ and modifications in latter studies^42,43^ and based on our complexes.^44^ The reaction rates were determined based on the formation of decamethylferrocenium cation (Fc*^+^). The selectivity for H_2_O_2_ (2e^–^/2H^+^ pathway) and H_2_O (4e^–^/4H^+^ pathway) was based on the final absorbance of Fc*^+^ determined by calculating the number of electrons transferred. For a complete 2e^–^/2H^+^ process, producing 100% H_2_O_2_, the maximum Fc*^+^ concentration would be 0.975 mM. For a complete 4e^–^/4H^+^ process producing 100% H_2_O, the maximum concentration of Fc*^+^ would be 1.95 mM. Control experiments conducted in the absence of O_2_ showed negligible Fc*^+^ formation, indicating that background HER was negligible under these experimental conditions. In contrast, significant Fc*^+^ formation was evident upon the introduction of O_2_, confirming the catalytic ORR (Figure S55). Additional control studies confirmed that in the absence of the Fe catalysts, Fc* alone is capable of reducing O_2_ due to its relatively low redox potential (Figures S58 and S59).^15^

### ORR Selectivity and Kinetics

The selectivity of each iron complex for H_2_O_2_ production was calculated from the plateau absorbance value of Fc*^+^. As depicted in Figure 1e, complex **1** showed a 79% selectivity to H_2_O_2_, consistent with literature values.^15^ Complex **2** had an 80% selectivity to H_2_O_2_, but with a slower kinetic profile as compared to **complex 1**. Complex **3** was the most selective for H_2_O_2_, indicating near quantitative preference (97%) for the 2e^–^/2H^+^ pathway, which is rare among heme and nonheme systems (Figure 1f).^45^ The H_2_O_2_ product yield catalyzed by complex **3** was further determined by iodometric and Ti­(O)­SO_4_ titration methods and found to be near quantitative. (Figures S51–S53).

Interestingly, the observed quantitative selectivity of **3** toward 2e^–^/2H^+^ chemistry aligns with our previous studies on selenate/selenite reduction, where increased O_2_ levels favored the formation of triphenylphosphine oxide.^46^ Here, H_2_O_2_ is likely formed in the presence of O_2_, which subsequently oxidizes triphenylphosphine to phosphine oxide.^46^

To elucidate the ORR mechanism of **3**, we conducted kinetic studies under pseudo-first-order conditions, varying the concentration of one reactant while keeping the others constant. As shown in Figure 2, we systematically examined the effects of (a) catalyst concentration, (b) acid concentration, (c) Fc* concentration, and (d) oxygen concentration at 298 K. The observed rate constant (*k*_obs_) was extracted from the plot of ln­(absorbance) vs *T* (Figures S25–S41). By graphing the initial rates against various reactant concentrations, an overall second-order reaction rate expression was derived, eq 1, whereas it is first order in catalyst and reductant yet zeroth order in oxygen and proton.

$$\mathrm{rate}=k_{\mathrm{cat}}[\mathrm{Fe}{]}^{1}[{\mathrm{Fc}}^{*}{]}^{1}[O_{2}{]}^{0}[H^{+}{]}^{0}$$ 1

To further investigate the reaction thermodynamics, we measured the *k*_cat_ values of the ORR at different temperatures to obtain an Eyring plot, enabling us to estimate entropy (Δ*S* ^‡^) and the reaction barrier Δ*G* ^‡^ (Δ*S* ^‡^ = −23.73 cal/mol/K, Δ*H* ^‡^ = 3.79 kcal/mol, Δ*G* ^‡^ = 10.86 kcal/mol, seeing Figure S48).

**Figure 2:**
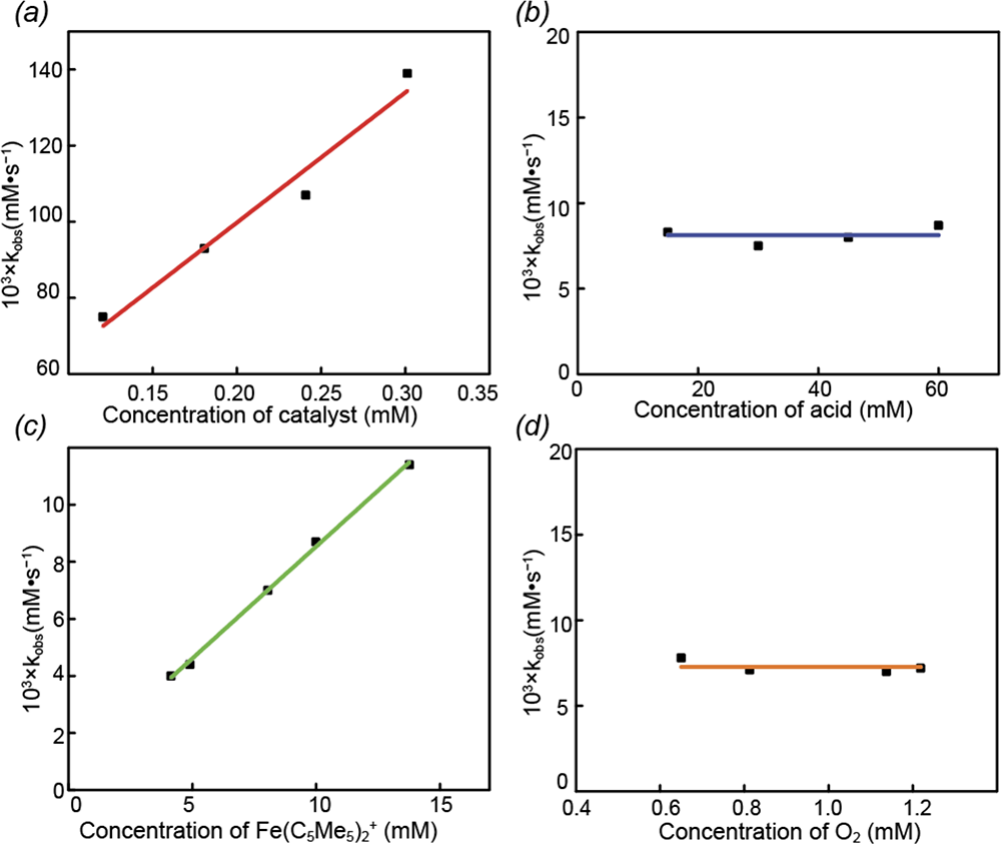
(a) Plot of *k*_obs_ vs [catalyst] in the presence of 0.4875 mM O_2_, 45 mM of HCl and 10 mM of Fc*. (b) Plot of *k*_obs_ vs [HCl] in the presence of 108 μM catalyst, 0.4875 mM O_2_, and 10 mM of Fc*. (c) Plot of *k*_obs_ vs [Fc*] in the presence of 110 μM catalyst, 0.4875 mM O_2_, and 45 mM of HCl. (d) Plot of *k*_obs_ vs [O_2_] in the presence of 110 μM catalyst, and 45 mM of HCl and 10 mM of Fc*.

### Proposed Catalytic Mechanism for **3**

Based on the experimental data, the catalytic mechanism for the 2e^–^/2H^+^ ORR is proposed, as illustrated in Scheme 2. The process begins with the reduction of the Fe^III^-oxo precatalyst to the active Fe^II^ species via a proton-coupled electron transfer (PCET), step A. The 1e^–^/2H^+^ reduction process is supported by UV–vis spectroscopy (Figure S54).

**Scheme 2:**
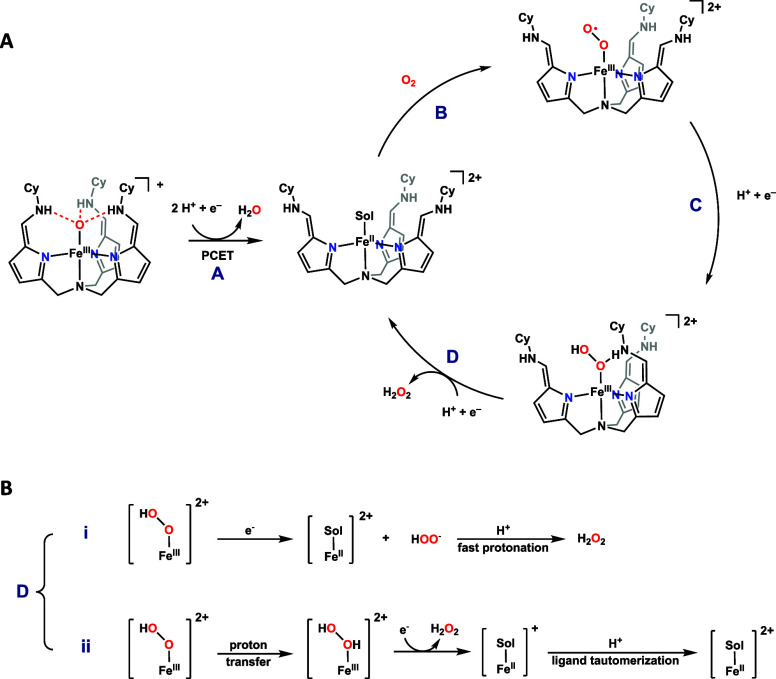
(A) Proposed Mechanism of ORR Catalyzed by **3**; (B) Two Possible Pathways on Step D

Subsequent coordination of molecular oxygen to the Fe^II^ center yields a Fe^III^-superoxide intermediate (Step B), which undergoes further proton and electron transfer to afford a putative Fe^III^–OOH intermediate (Step C). A final reduction and protonation step yields hydrogen peroxide and regenerates the Fe^II^ active site (Step D). Kinetic studies indicate a first-order dependence on both the reductant and the catalyst, suggesting that Step D likely proceeds through a stepwise proton transfer–electron transfer (PTET) or electron transfer–proton transfer (ETPT) pathway, with the electron transfer step being rate-limiting. While both pathways are chemically plausible, electrostatic repulsion between the cationic Fe^III^–OOH species and an incoming proton disfavors initial protonation. Therefore, an ETPT pathway is proposed to be more favorable.

Two mechanistic alternatives are considered for the ETPT pathway (Scheme 2B):^47,48^

1. HOO^–^ dissociated from the intermediate Fe^III^–OOH, followed by protonation generate H_2_O_2_.
2. HOO^–^ remains coordinated to the iron center and is protonated via a hydrogen-bonding network within the secondary coordination sphere. Electron transfer facilitates H_2_O_2_ release, accompanied by the formation of a mixed-arm Fe^II^ species. Protonation-induced tautomerization of the ligand backbone regenerates the active Fe^II^ catalyst.

Although polar solvents such as DMF have been shown to facilitate HOO^–^ dissociation in porphyrin systems,^48^ we hypothesize in this system that pathway (ii) is more favorable. First, the hydrogen-bonding framework in the secondary coordination sphere is expected to stabilize either HOO^–^ or H_2_O_2_,^49^ consistent with systems that bind even weakly coordinating anions such as nitrate, perchlorate, and selenate.^46,50–52^ Second, ligand framework tautomerization provides a flexible proton relay network, enabling intramolecular proton transfer, where the ligand could be reprotonated to regenerate the active Fe^II^ catalyst. This mechanistic proposal is consistent with prior studies on Fe–porphyrins, where H-bonding interactions have been shown to facilitate protonation of Fe–OOH species and promote H_2_O_2_ release.^47^ Further computational and experimental investigations are currently underway to better define the intermediates and reaction routes involved in this catalytic cycle.

Additionally, the possibility of a bimetallic Fe–O–O–Fe intermediate was excluded. Our previous studies of the tripodal Fe^III^ oxygen species with different ligand architectures suggest that bridged Fe-oxo-Fe dimers readily form only when fewer than two ligand arms are present. Since the current system has three ligand arms, the dinuclear pathway is unlikely to contribute to the proposed catalytic pathway.

Most nonheme iron complexes, previously reported in the literature, featuring secondary coordination sphere hydrogen bonding, have often been implicated in promoting O_2_ binding and O–O bond cleavage, leading to H_2_O as the major product. In contrast, our tripodal Fe^III^ complex exhibits high selectivity for H_2_O_2_ formation, likely due to the stabilization of the Fe^III^ hydroperoxide intermediate, distinguishing it from those previously reported.

## Conclusions

We have investigated the oxygen activation reactivity of our tetrapodal and tripodal iron complexes **2** and **3**, using both electrochemical methods and external chemical reductants. For comparison, catalytic studies were also conducted with porphyrin complex **1**. While **2** demonstrated selectivity similar to that of **1** but slower kinetics, **3** exhibited remarkable (∼97%) selectivity for the two-electron/two-proton reduction pathway. To gain a deeper understanding of the mechanism, we performed concentration-dependent oxygen activation studies, which suggested that the catalytic reaction follows second-order kinetics, being first-order with respect to both the reductant and the catalyst concentrations.

Ongoing investigations, incorporating both experimental and theoretical approaches, seek to further elucidate the mechanistic role of polypodal iron complexes, particularly the influence of secondary coordination sphere hydrogen bonding. Future studies will explore the effects of various ligand modifications, including electron-withdrawing and electron-donating groups in the second coordination sphere, as well as variations in the number of hydrogen-bond donors.^44,53,54^

## Supplementary Material



## Notes

Experimental details and spectra (PDF)
