# Supplementary material for: Nonheme Iron Catalyst Selectively Activates Oxygen to Hydrogen Peroxide
Source: JACS Au. 2025 Jun 11;5(6):2713–9. doi: 10.1021/jacsau.5c00320 (PMC12188414; doi:10.1021/jacsau.5c00320)
Supplement: Supplementary file 1 [file au5c00320_si_001.pdf]

Non-Heme Iron Catalyst Selectively Activates Oxygen to Hydrogen Peroxide

Hsien-Liang Cho,^[a] Daoyang Zhang,^[a] and Alison R. Fout*[a]

[a]Department of Chemistry, Texas A&M University, 580 Ross St. College Station, Texas 77843, USA.,

E-mail for A.R.F.: fout@tamu.edu.

authors contributed equally, listed alphabetically

Supplementary Information

Contents

General considerations: 3

Materials and Methods 3

Physical methods. 3

Synthesis of metal complexes 4

Synthesis of FeTPPCl 4

Synthesis of $N(afa^{Cy})_3Fe(O)OTf$ 4

Synthesis of $[Py_2Py(afa^{Cy})_2Fe(OH)]OTf_2$ 4

Synthesis of decamethylferrocenium chloride (Fc*Cl) 4

^1H NMR spectroscopy 5

General Conditions for kinetic studies. 7

Calibration curve for decamethylferrocenium chloride. 7

Chemical reduction reaction rates studies with various equivalents of reagents and temperatures: 7

Spectroscopic determination of product selectivity and reaction order. 8

Mechanistic investigation for generating active $N(afa^{Cy})_3FeOTf_2$ 8

UV-Vis spectroscopy 8

Calculation of kinetic parameters. 30

Titration experiments for H_2O_2 determination. 32

Iodometric titration. 32

$Ti(O)SO_4$ titration. 33

Electrochemistry measurements 36
Reference: 37

General considerations:

Materials and Methods.

The synthesis of porphyrin iron complex was done under ambient conditions. Both nonheme tripodal and tetrapodal iron complexes were carried out in the absence of water and dioxygen, due to the air and moisture sensitivity of the compounds, using a Vigor inert atmosphere glovebox under a nitrogen atmosphere unless otherwise specified. For the glovebox reaction, all glassware was dried in an oven for at least 4 h and cooled in an evacuated antechamber prior to use. Solvent was dried and deoxygenated on a Vigor Solvent Purification System and stored over 3 Å molecular sieves, purchased from Sigma-Aldrich, prior to use. Deuterated solvents were purchased from Cambridge Isotope Laboratories and stored over 3 Å molecular sieves prior to use. Celite 545 (J. T. Baker) was heated to 150 °C under dynamic vacuum for 24 h prior to use in the drybox. All reagents were purchased from commercial sources and used as received unless otherwise noted.

Physical methods.

^{1}H NMR spectra were recorded at ambient temperature on a Bruker Avance Neo console operating at 400 MHz for ^{1}H NMR spectra. Ultraviolet−visible (UV−Vis) spectroscopy was performed on an Agilent Technologies Cary Series UV−Vis NIR 5000 spectrometer. UV−Vis spectra were recorded on an Agilent 8453 spectrophotometer with accompanying software. All samples were prepared under Schlenk's line containing a dinitrogen atmosphere in quartz cuvettes with a 1 cm path length and capped with a rubber septum. All electrochemical experiments were performed using an CHI bipotentiostat. All electrochemical experiments were performed with a glassy carbon working electrode, a Pt counter electrode, an Ag wire reference electrode, 0.1 M tetrabutylammonium hexafluorophosphate ($TBAPF_6$), and acetonitrile solvent. $TBAPF_6$ was recrystallized three times with 200-proof ethanol and dried at least for 24 hours under high vacuum prior to use. A spatula-tip quantity of ferrocene (FcH) was added after initial data was collected and used as an internal standard by referencing to the $FcH^{+/0}$ couple.

Synthesis of metal complexes

Synthesis of FeTPPCl

The molecule was prepared by following a literature procedure with slight modification.[1, 2] A solution of tetraphenylporphyrin (0.100 g, 0.163 mmol) in DMF (25 mL) was stirred in a round bottom flask and brought to reflux. $FeCl_2 \cdot 4H_2O$ (0.160 g, 0.805 mmol, 5 equivalents) was then added to the reaction flask as a solid. The reflux was continued overnight. The crude mixture was then cooled, and the solvent was removed under reduced pressure, resulting in a dark solid. The dark solid was suspended in chloroform (~30 mL), washed with brine (3 × 30 mL), and dried over Na_2SO_4. The solution was filtered and concentrated under reduced pressure to ~7 mL. Excess hexane (~20 mL) was layered to slow;y precipitate a solid that was collected by centrifugation and washed with hexane to yield the product as a crystalline dark brown solid (yield: 0.078 g, 76%). ^{1}H NMR spectrum matched to the previous literature.[1, 2]

Synthesis of N(afa^{Cy})$_3$Fe(O)OTf

The synthesis of N(afa^{Cy})$_3$FeOTf$_2$ was followed previous procedure.[3] To a 20 mL scintillation vial were added N(afa^{Cy})$_3$FeOTf$_2$ (0.054 g, 0.058 mmol), two drops of triethylamine (~10 mg), and 2 mL of acetonitrile. The reaction was brought outside the glovebox and stirred for 10 minutes. Upon exposure to air, the immediate color change from light yellow to dark brown was observed. The volatiles were then removed under reduced pressure. The resulting precipitate was recrystallized from acetonitrile and ether twice to form a target product (0.039 g, 0.049 mmol, 84%). ^{1}H NMR spectrum matched to the previous literature.[4]

Synthesis of [Py$_2$Py(afa^{Cy})$_2$Fe(OH)]OTf$_2$

The synthesis of [Py$_2$Py(afa^{Cy})$_2$Fe]OTf$_2$ was followed previous procedure.[5] To a 20 mL scintillation vial were added [Py$_2$Py(afa^{Cy})$_2$Fe]OTf$_2$ (0.038 g, 0.038 mmol), $KBrO_3$ (0.006 g, 0.038 mmol), and 4 mL of acetonitrile. The reaction was stirred overnight, concomitant with the color change from bright yellow to dark brown. The volatiles were then removed under reduced pressure. The resulting precipitate was dissolved in dichloromethane (6 mL) and filtered over a pad of celite. The dichloromethane filtrate was dried in vacuo, forming a dark-brown powder (0.037 g, 0.037 mmol, 97%). ^{1}H NMR spectrum matched to the previous literature.[5]

Synthesis of decamethylferrocenium chloride (Fc*Cl)

Under ambient conditions, $PhICl_2$ (0.070 g, 0.26 mmol) dissolved in 1 mL of acetonitrile was added to a decamethylferrocene (0.113 g, 0.35 mmol) in 1 mL of acetonitrile solution. The solution was stirred for 1 hour, during which the color changed from yellow to dark green. The solvent was then removed under reduced pressure. The resulting dark green solid was washed with THF (6 mL) and further eluted with acetonitrile (8 mL) to isolate the desired product. The volatiles were removed under vacuum, yielding a dark green powder (0.098 g, 0.027 mmol, 77%). ^{1}H NMR (400 MHz, 298 K, CD_3CN) δ 36.97.

^{1}H NMR spectroscopy

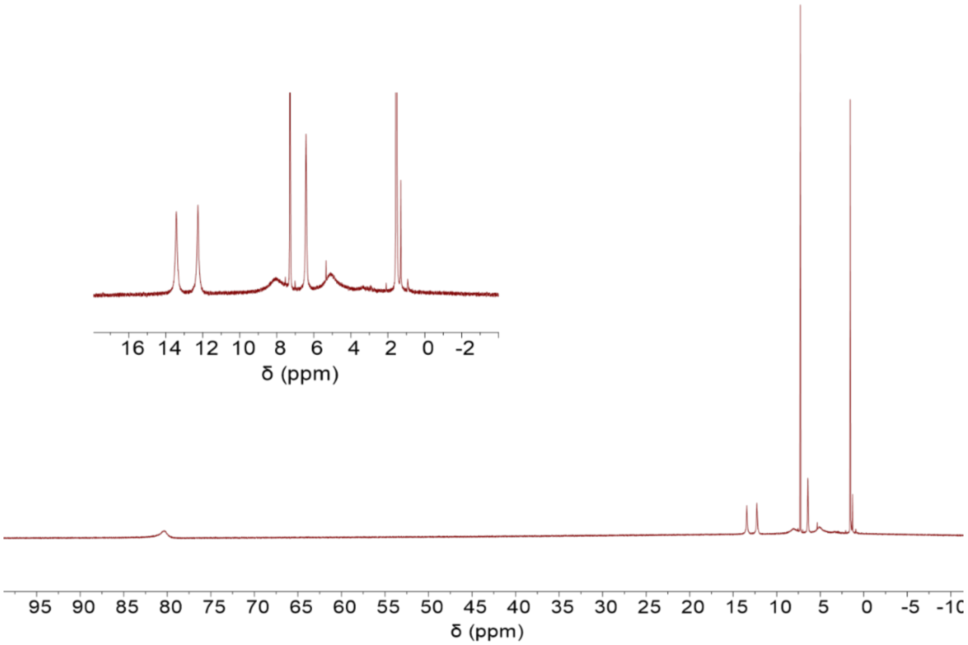

Figure S1. ^{1}H NMR spectrum of TPPFeCl ($CDCl_3$, 400 MHz, 298 K).

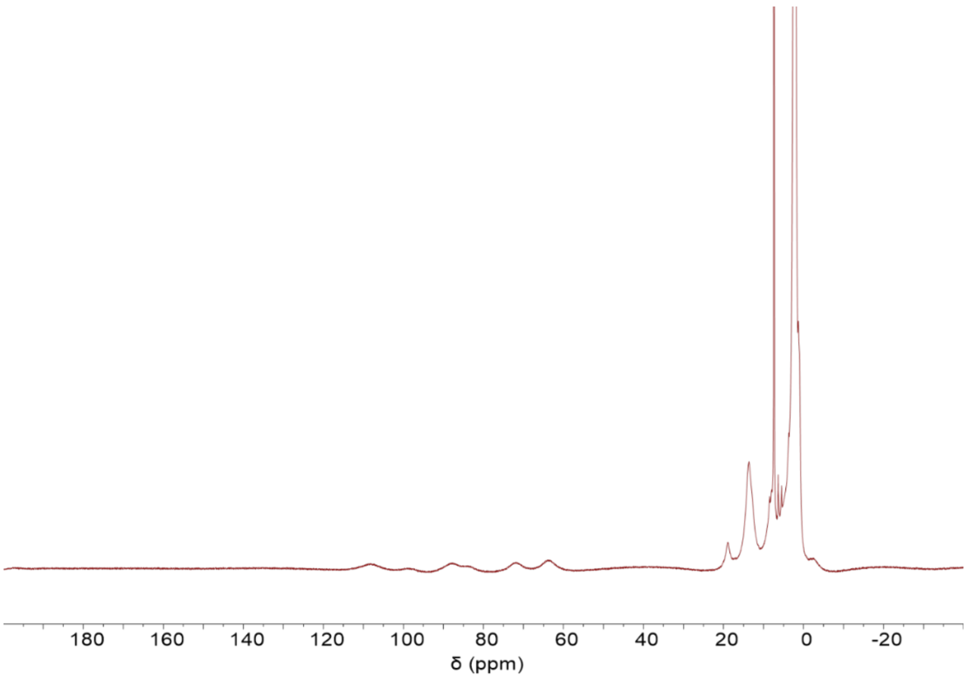

Figure S2. ^{1}H NMR spectrum of $[Py_2Py(afa^{Cy})_2Fe(OH)]OTf_2$ (CD_3CN, 400 MHz, 298 K).

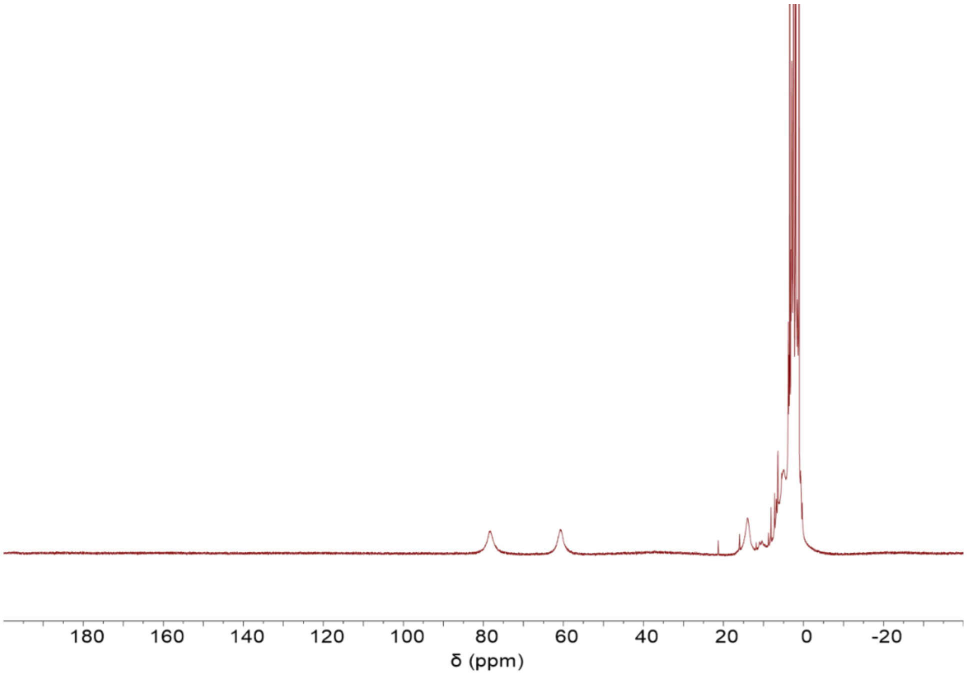

Figure S3. ^{1}H NMR spectrum of N(afa^{Cy})$_3$Fe(O)OTf (CD$_3$CN, 400 MHz, 298 K).

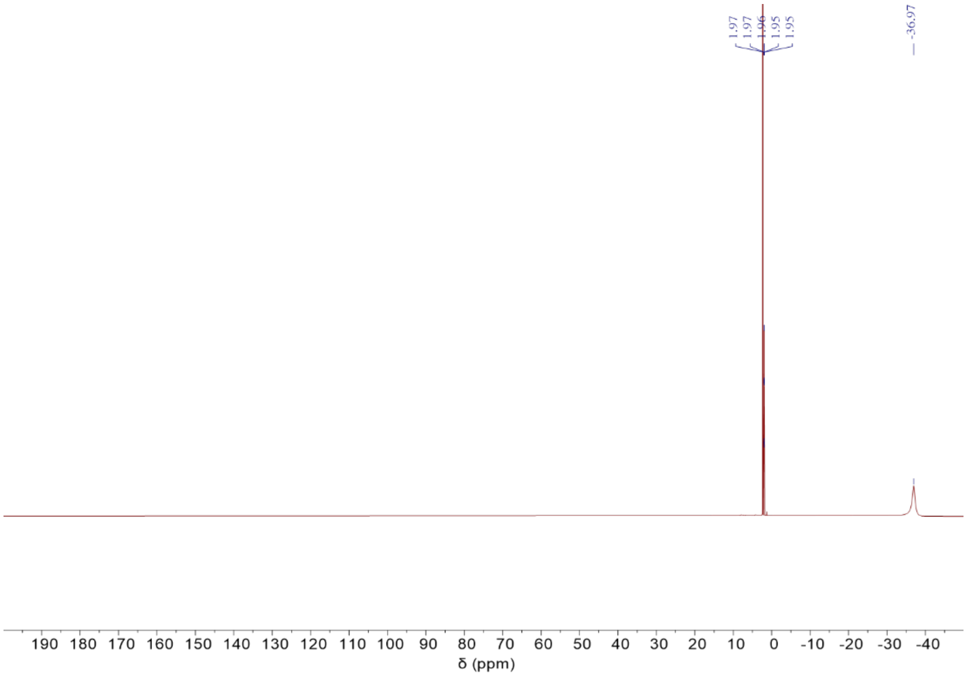

Figure S4. ^{1}H NMR spectrum of decamethylferrocenium chloride (CD$_3$CN, 400 MHz, 298 K).

General Conditions for kinetic studies.

Calibration curve for decamethylferrocenium chloride.

A solvent mixture of benzonitrile (PhCN) and dimethylformamide (DMF) in a 1:3 ratio was prepared for the calibration curve. To prepare the stock solution, 7.7 mg of decamethylferrocenium chloride was weighed and dissolved in a 2.0 mL volumetric flask, yielding a concentration of 10.6 mM. A 0.4 mL aliquot of the stock solution was transferred into a cuvette, followed by the addition of 1.6 mL of the solvent mixture. The absorbance of this solution was measured, and subsequent measurements were taken after adding 0.3 mL of the mixed solvent. This process was repeated three times. Then, 1.0 mL of the solution was removed, and the same procedure was followed by adding 0.3 mL of the mixed solvent for the additional four measurements.

Chemical reduction reaction rates studies with various equivalents of reagents and temperatures:

In the Schlenk's line, PhCN was bubbled with N_2, and DMF was bubbled with air for a minimum of 15 minutes before use. The O_2 concentrations in air-saturated (0.65 mM) and O_2-saturated (3.25 mM) DMF solutions were known from the literature.[1, 6] Iron catalysts and decamethylferrocene were weighed into separate scintillation vials and then diluted to a final volume of 3.0 mL to prepare stock solutions. These solutions were purged with N_2 for 10 minutes to avoid any O_2 present. To initiate the reaction, 0.5 mL of the iron catalyst solution and 0.5 mL of the decamethylferrocene solution were transferred into a quartz cuvette equipped with a rubber septum. 3.0 mL of the DMF solution was added. This mixture was used as the background under controlled temperatures and stirring rates. Subsequently, HCl was injected into the cuvette and the reaction was monitored continuously for 10 minutes with measurements taken every 0.1 or 0.5 seconds. The reaction order was determined via comparisons of the reaction rate among reagents equivalents.

For reduction experiments run under Fc*-limiting condition, 3.0 mL of O_2-saturated DMF, 1.0 mL of O_2-saturated PhCN containing 1.9 mM Fc, 265 μM of the complex **3**, and 45 mM HCl were mixed together in a quartz cuvette. Iodometric titrations were conducted to determine the amount of H_2O_2 produced after all the Fc* was exhausted and converted to Fc^{*+}.

Spectroscopic determination of product selectivity and reaction order.

The disappearance rate of Fc* is the same as the formation rate of Fc^{*+}. By changing different concentrations of each reagents and maintaining the reaction under pseudo first order conditions, the orders, m, n, x, and y, could be determined after plotting $\ln\left(\frac{[Fc^{*+}]_{\infty}-[Fc^{*+}]_0}{[Fc^{*+}]_{\infty}}\right)$ vs. time.

$$rate = \frac{d[Fc^{*}]}{dt} = -\frac{d[Fc^{*+}]}{dt} = -k_{cat}[cat]^m[H^+]^x[O_2]^n[Fc^*]^y$$

$$ln\left(\frac{[Fc^*]}{[Fc^*]_0}\right) = \ln\left(\frac{[Fc^{*+}]_{\infty} - [Fc^{*+}]_0}{[Fc^{*+}]_{\infty}}\right) = -k_{obs}t = -k_{cat}[cat]^m[H^+]^x[O_2]^n t$$

Mechanistic investigation for generating active $N(afa^{Cy})_3FeOTf_2$

$N(afa^{Cy})_3Fe(O)OTf$ (1.7 mg, 0.002 mmol) and decamethylferrocene (0.7 mg, 0.002 mmol) were weighed into the same scintillation vial. A 1:1 mixture of tetrahydrofuran (THF) and acetonitrile (MeCN) was prepared, and 4.0 mL of this mixed solvent was added to the vial. THF was included to improve the solubility of decamethylferrocene. The resulting reaction solution was equally divided between two separate cuvettes, each diluted to a final volume of 3.0 mL. To one cuvette, 2.4 equivalents of trifluoroacetic acid (TFA) were added, while the other remained without TFA.

UV-Vis spectroscopy

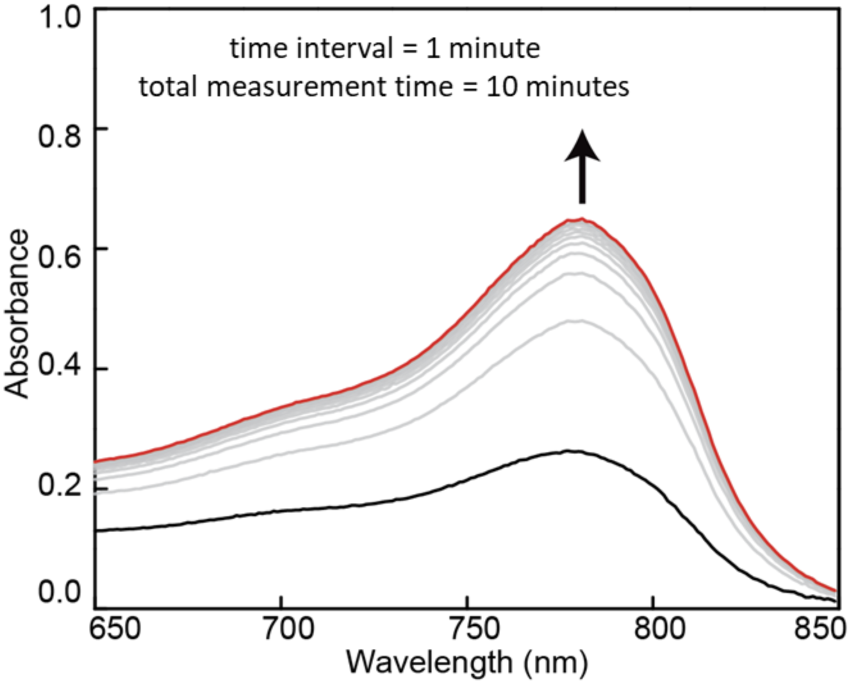

Figure S5. Absorption spectrum recorded for oxygen activation chemical reduction with $N(afa^{Cy})_3Fe(O)OTf$. The peak indicates the formation of decamethylferrocenium ion, λ_{max}= 780 nm. (Reaction conditions: 250 µM of catalyst, 45 mM of HCl, 10 mM of decamethylferrocene, and , 0.4875 mM O_2 in 4 mL mixed PhCN and DMF solution in a gas-tight cuvette.)

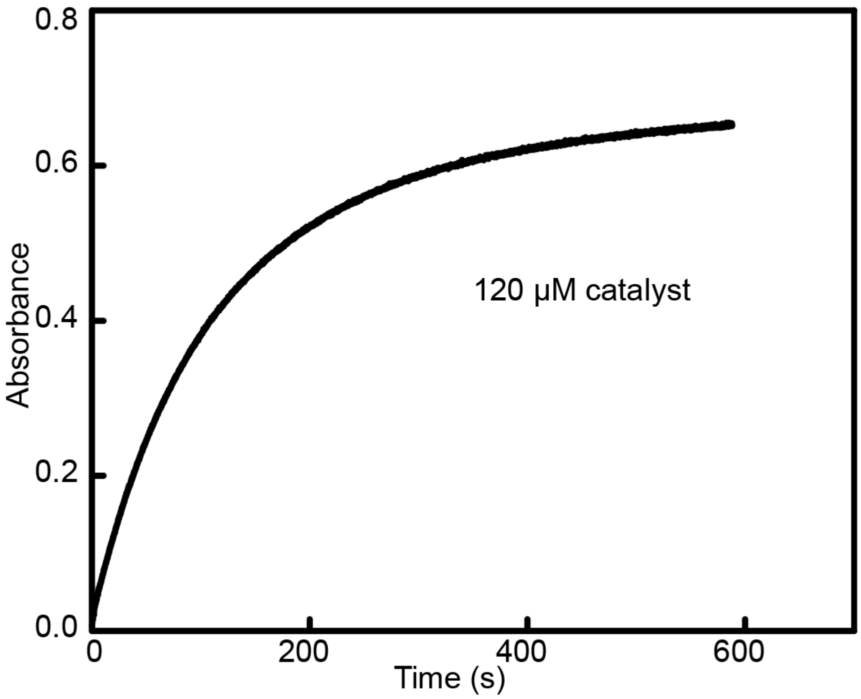

Figure S6. Plot absorbance at 780 nm vs. time in the presence of 120 µM of catalyst, 0.4875 mM O_2, 45 mM of HCl and 10 mM of decamethylferrocene. $N(afa^{Cy})_3Fe(O)OTf$ was used as catalyst.

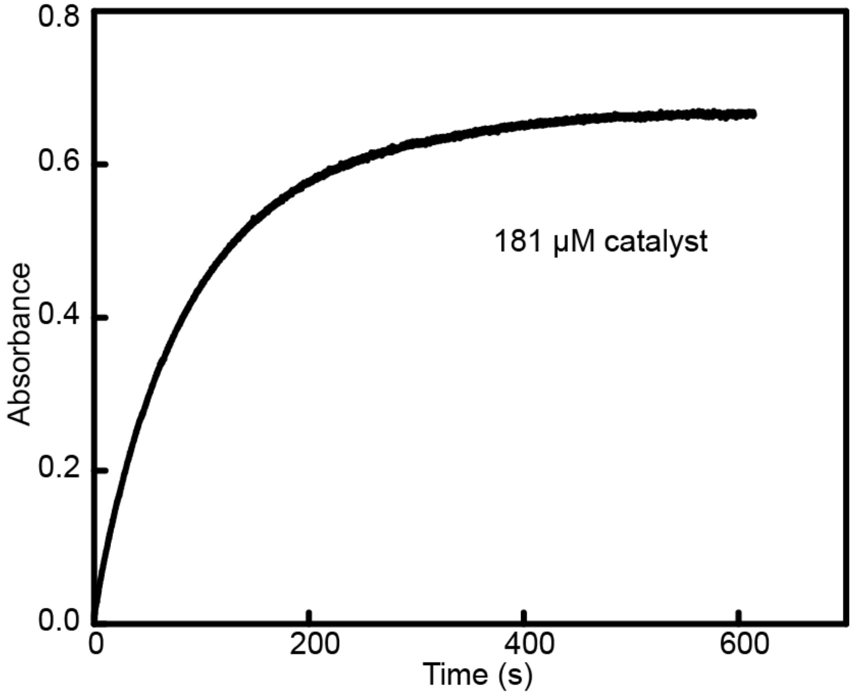

Figure S7. Plot absorbance at 780 nm vs. time in the presence of 181 µM of catalyst, 0.4875 mM O_2, 45 mM of HCl and 10 mM of decamethylferrocene. $N(afa^{Cy})_3Fe(O)OTf$ was used as catalyst.

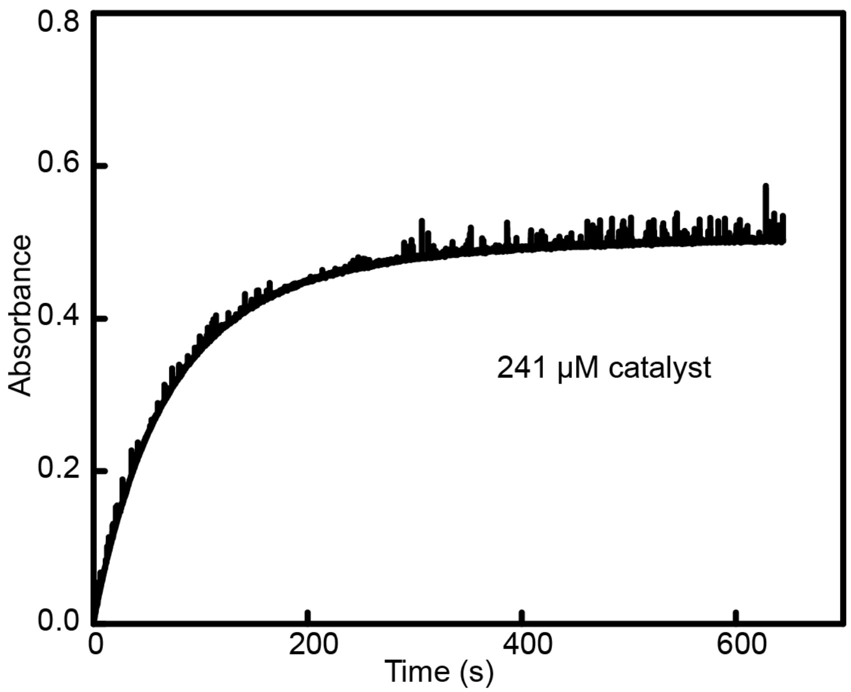

Figure S8. Plot absorbance at 780 nm vs. time in the presence of 241 μM of catalyst, 0.4875 mM O_2, 45 mM of HCl and 10 mM of decamethylferrocene. $N(afa^{Cy})_3Fe(O)OTf$ was used as catalyst.

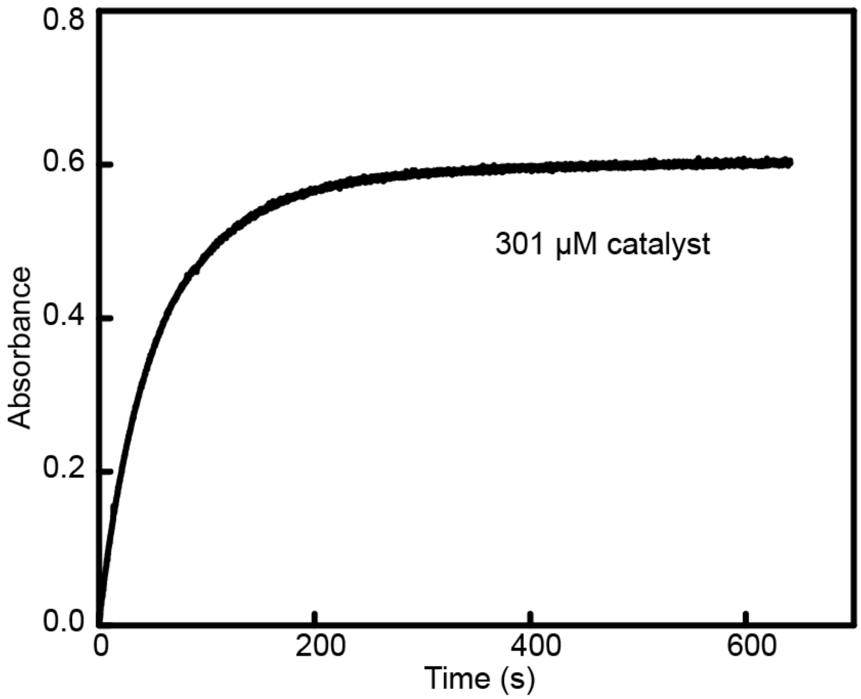

Figure S9. Plot absorbance at 780 nm vs. time in the presence of 301 μM of catalyst, 0.4875 mM O_2, 45 mM of HCl and 10 mM of decamethylferrocene. $N(afa^{Cy})_3Fe(O)OTf$ was used as catalyst.

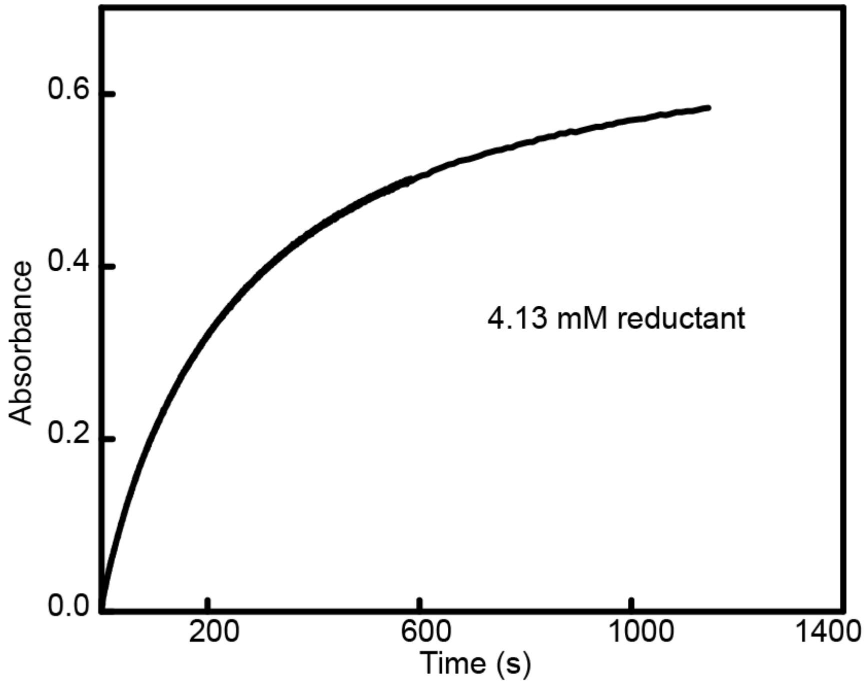

Figure S10. Plot absorbance at 780 nm vs. time in the presence of 108 μM catalyst, 0.4875 mM O_2, 45 mM of HCl, and 4.13 mM of decamethylferrocene. N(afa^{Cy})$_3$Fe(O)OTf was used as catalyst.

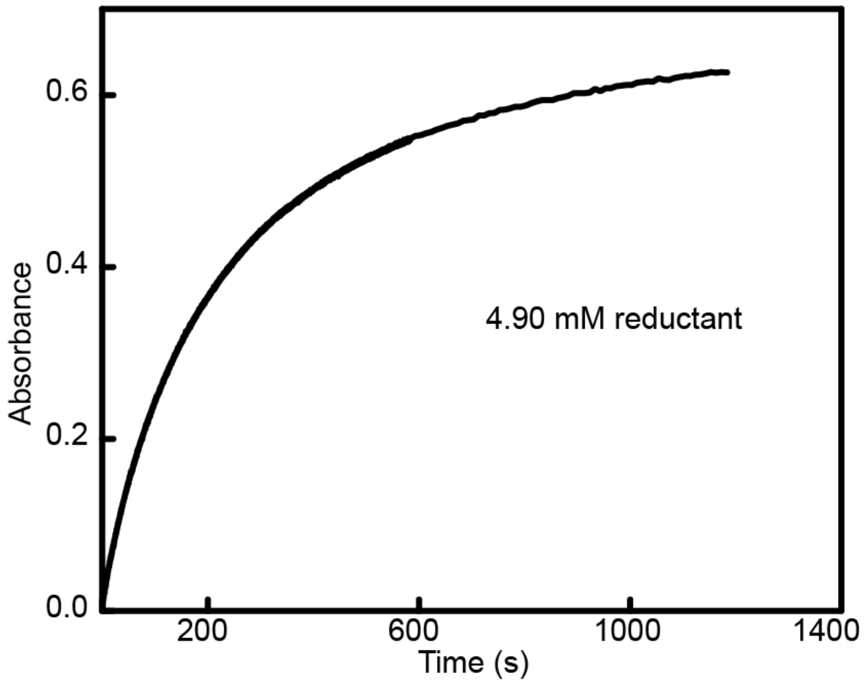

Figure S11. Plot absorbance at 780 nm vs. time in the presence of 110 μM catalyst, 0.4875 mM O_2, 45 mM of HCl, and 4.90 mM of decamethylferrocene. N(afa^{Cy})$_3$Fe(O)OTf was used as catalyst.

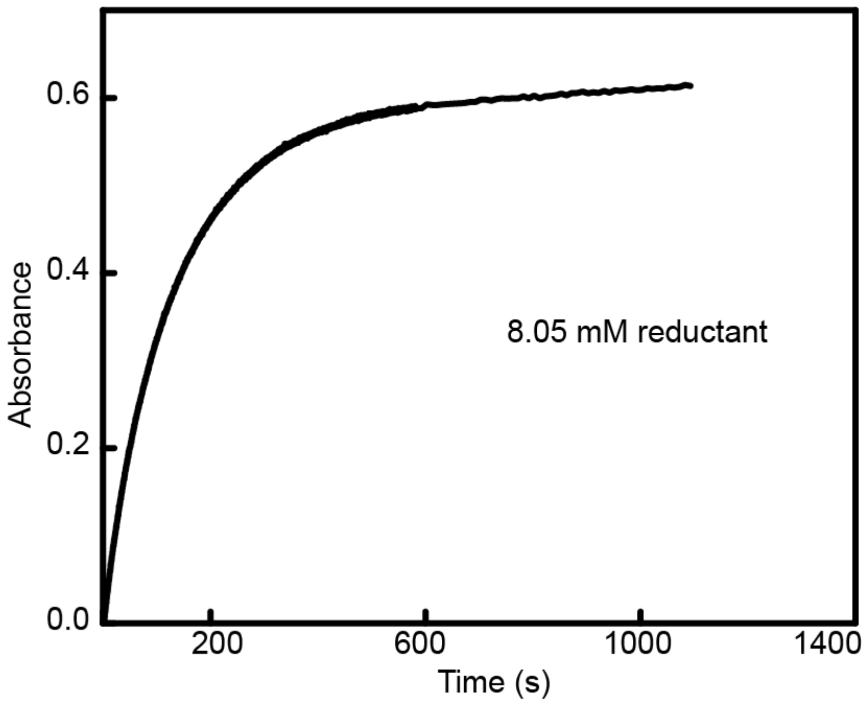

Figure S12. Plot absorbance at 780 nm vs. time in the presence of 110 μM catalyst, 0.4875 mM O_2, 45 mM of HCl, and 8.05 mM of decamethylferrocene. N(afa^{Cy})$_3$Fe(O)OTf was used as catalyst.

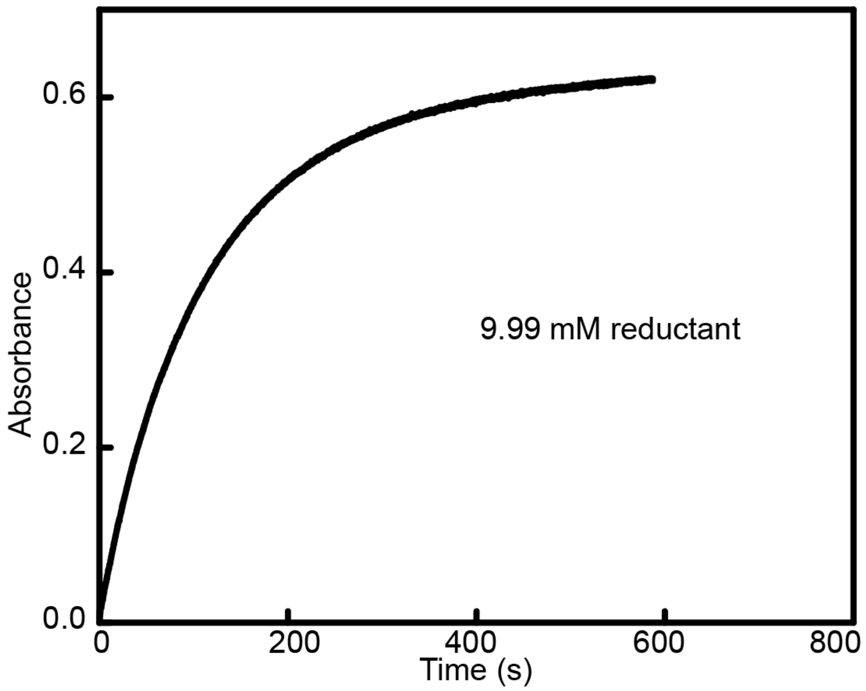

Figure S13 Plot absorbance at 780 nm vs. time in the presence of 110 μM catalyst, 0.4875 mM O_2, 45 mM of HCl, and 9.99 mM of decamethylferrocene. N(afa^{Cy})$_3$Fe(O)OTf was used as catalyst.

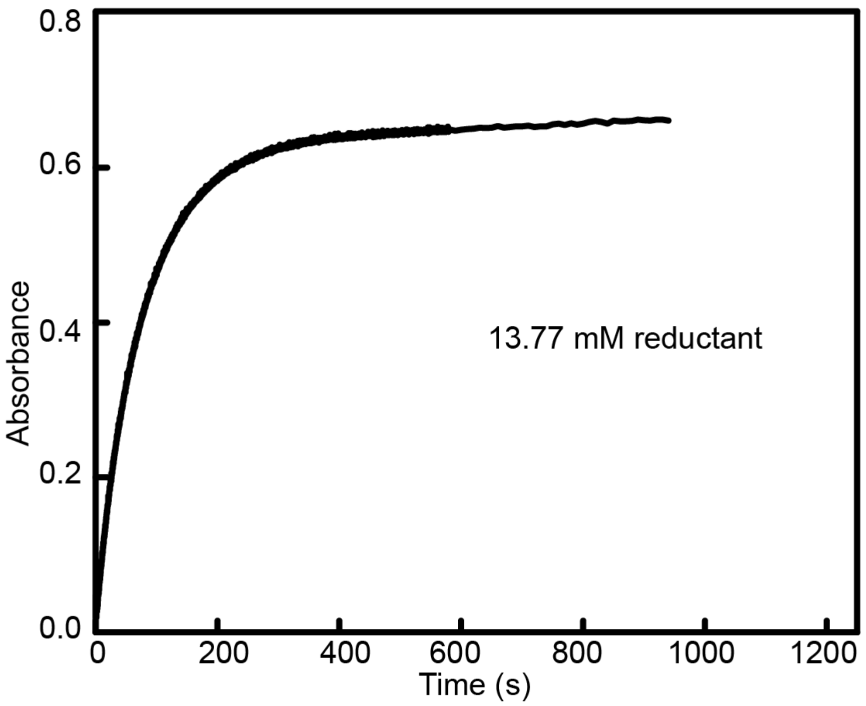

Figure S14. Plot absorbance at 780 nm vs. time in the presence of 110 µM catalyst, 0.4875 mM O_2, 45 mM of HCl, and 13.77 mM of decamethylferrocene. N(afa^{Cy})$_3$Fe(O)OTf was used as catalyst.

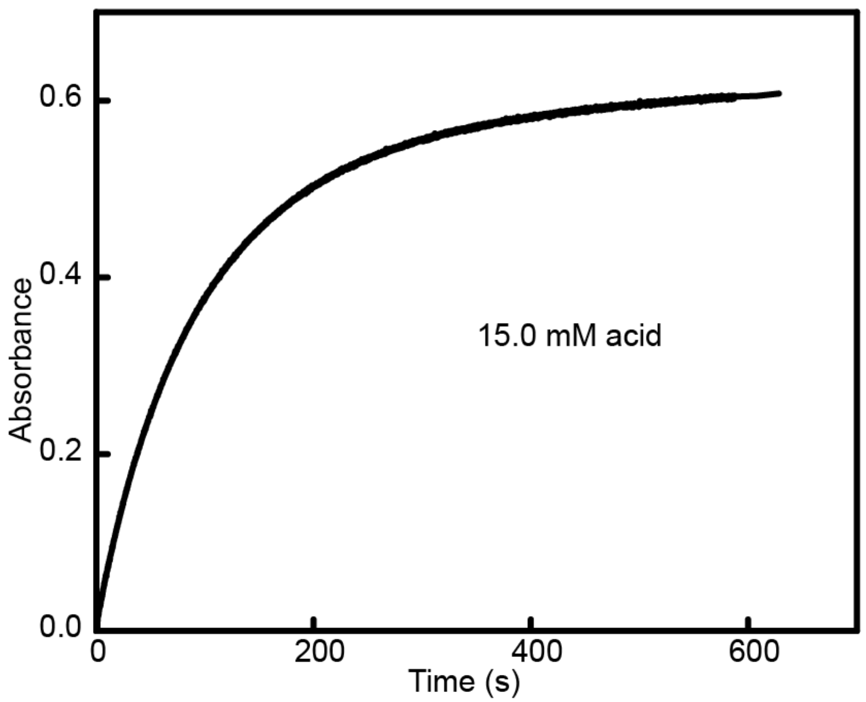

Figure S15. Plot absorbance at 780 nm vs. time in the presence of 108 µM catalyst, 0.4875 mM O_2, 15.0 mM of acid, and 10 mM of decamethylferrocene. N(afa^{Cy})$_3$Fe(O)OTf was used as catalyst.

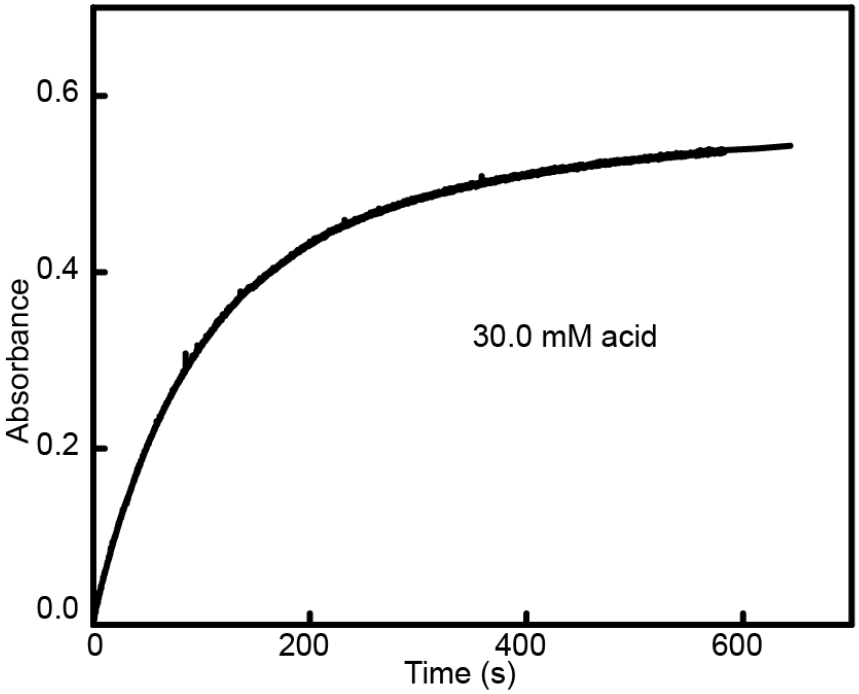

Figure S16. Plot absorbance at 780 nm vs. time in the presence of 108 μM catalyst, 0.4875 mM O_2, 30.0 mM of acid, and 10 mM of decamethylferrocene. N(afa^{Cy})$_3$Fe(O)OTf was used as catalyst.

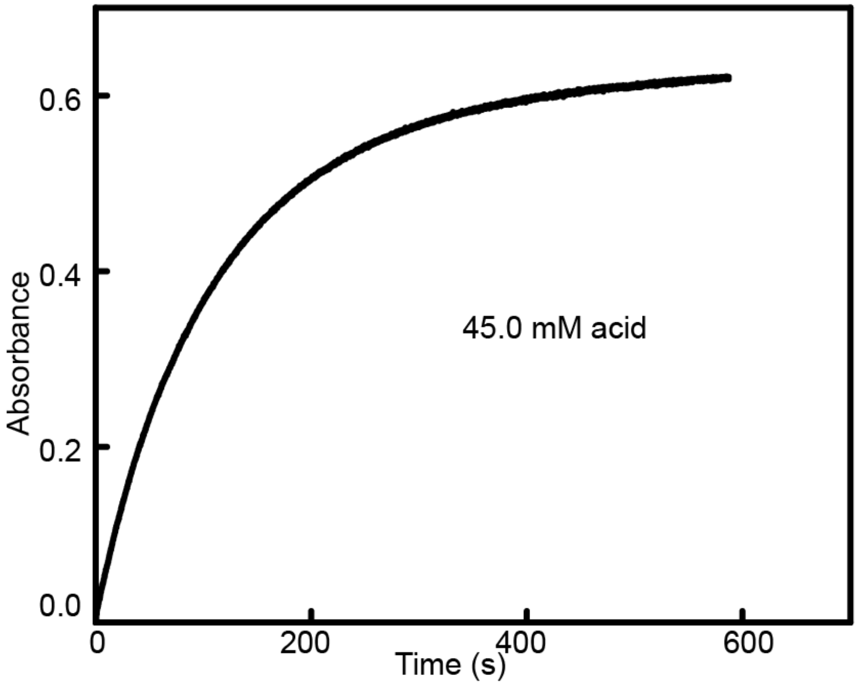

Figure S17. Plot absorbance at 780 nm vs. time in the presence of 108 μM catalyst, 0.4875 mM O_2, 45.0 mM of acid, and 10 mM of decamethylferrocene. N(afa^{Cy})$_3$Fe(O)OTf was used as catalyst.

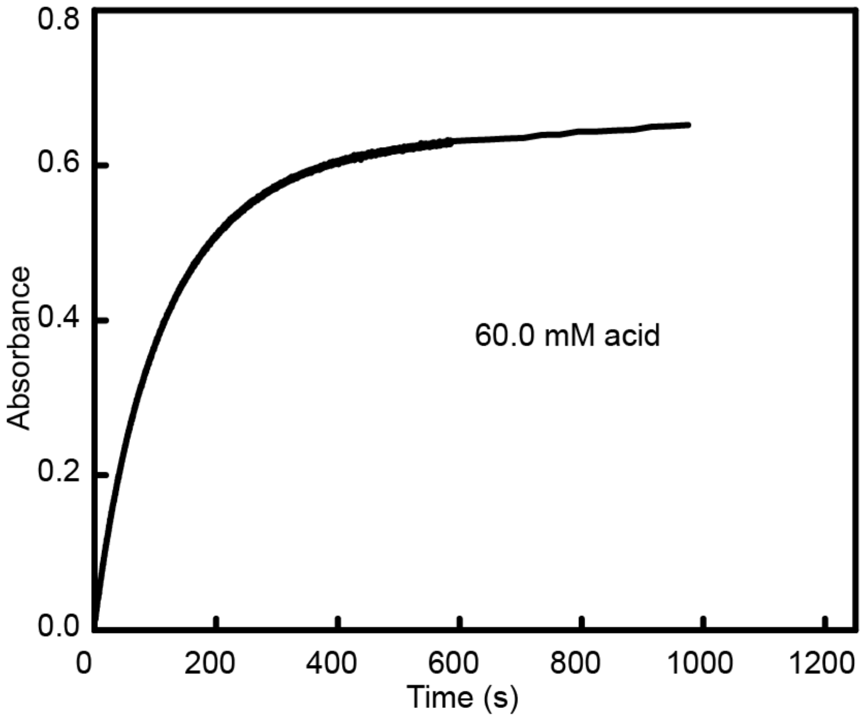

Figure S18. Plot absorbance at 780 nm vs. time in the presence of 108 μM catalyst, 0.4875 mM O_2, 60.0 mM of acid, and 10 mM of decamethylferrocene. N(afa^{Cy})$_3$Fe(O)OTf was used as catalyst.

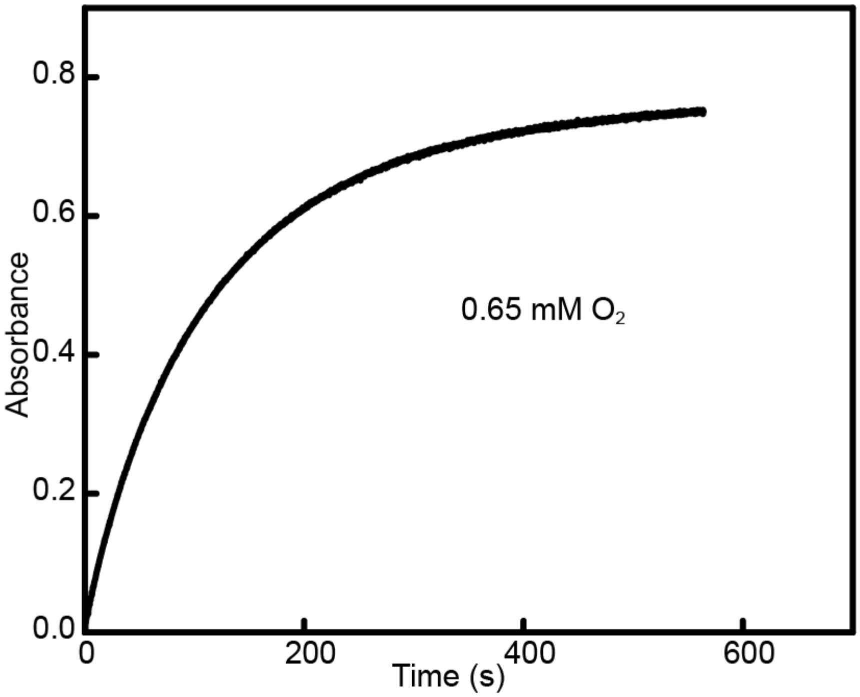

Figure S19. Plot absorbance at 780 nm vs. time in the presence of 110 μM catalyst, 0.65 mM O_2, 45 mM of HCl and 10 mM of decamethylferrocene. N(afa^{Cy})$_3$Fe(O)OTf was used as catalyst.

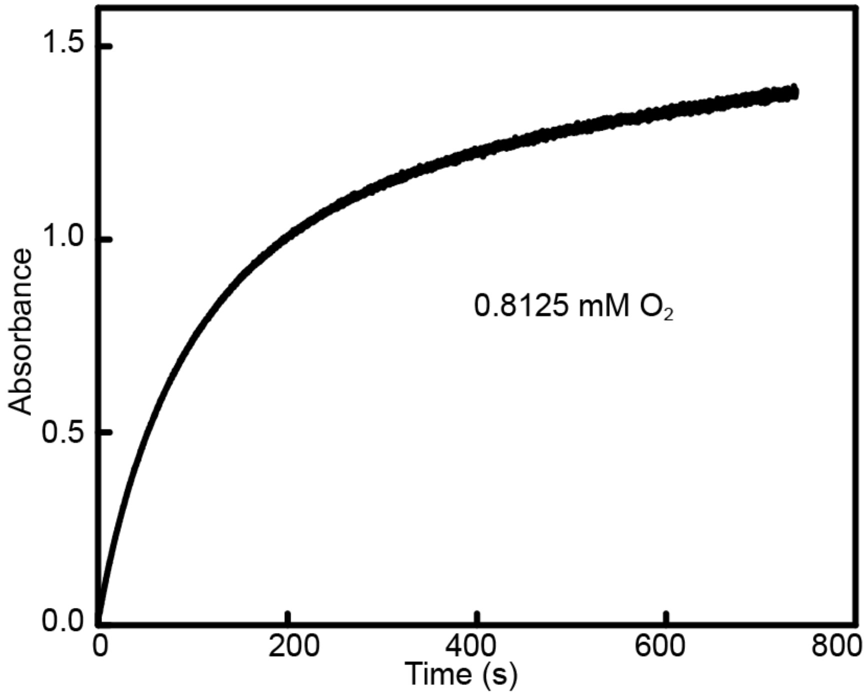

Figure S20. Plot absorbance at 780 nm vs. time in the presence of 110 μM catalyst, 0.8125 mM O_2, 45 mM of HCl and 10 mM of decamethylferrocene. N(afa^{Cy})$_3$Fe(O)OTf was used as catalyst.

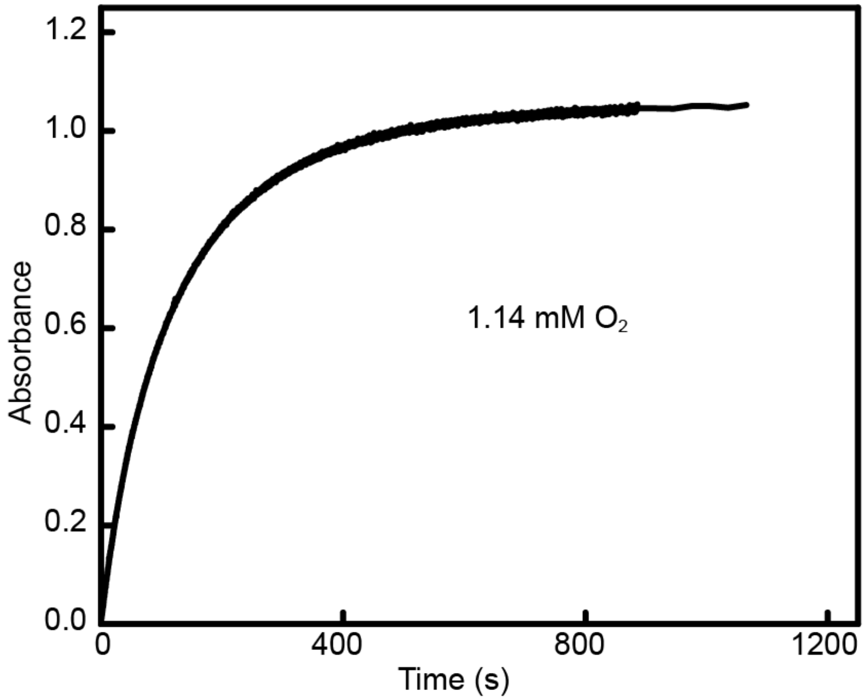

Figure S21. Plot absorbance at 780 nm vs. time in the presence of 110 μM catalyst, 1.14 mM O_2, 45 mM of HCl and 10 mM of decamethylferrocene. N(afa^{Cy})$_3$Fe(O)OTf was used as catalyst.

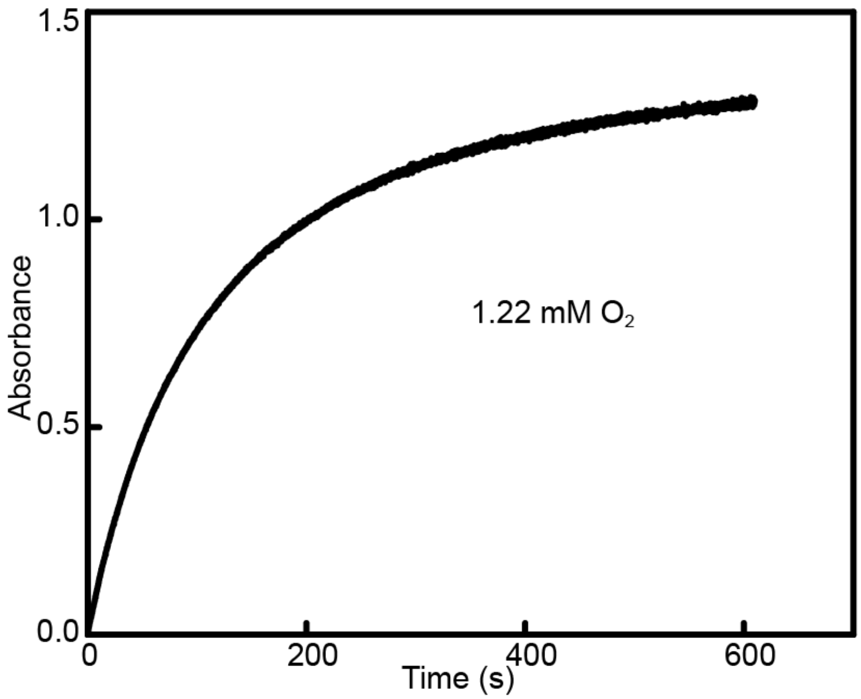

Figure S22. Plot absorbance at 780 nm vs. time in the presence of 110 μM catalyst, 1.22 mM O_2, 45 mM of HCl and 10 mM of decamethylferrocene. N(afa^{Cy})$_3$Fe(O)OTf was used as catalyst.

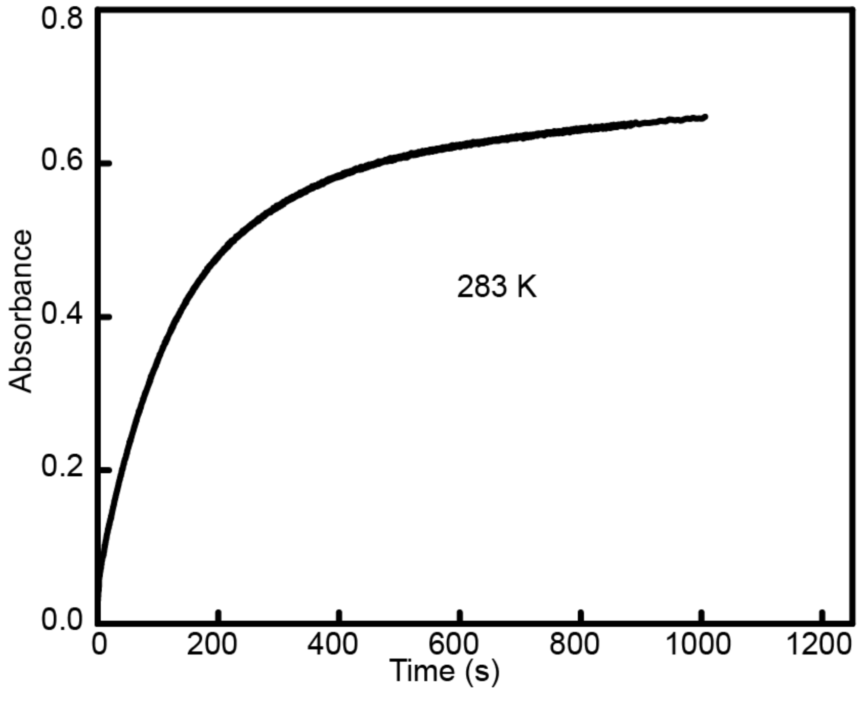

Figure S23. Plot absorbance at 780 nm vs. time in the presence of 110 μM catalyst, 0.4975 mM O_2, 45 mM of HCl and 10 mM of decamethylferrocene at 283 K. N(afa^{Cy})$_3$Fe(O)OTf was used as catalyst.

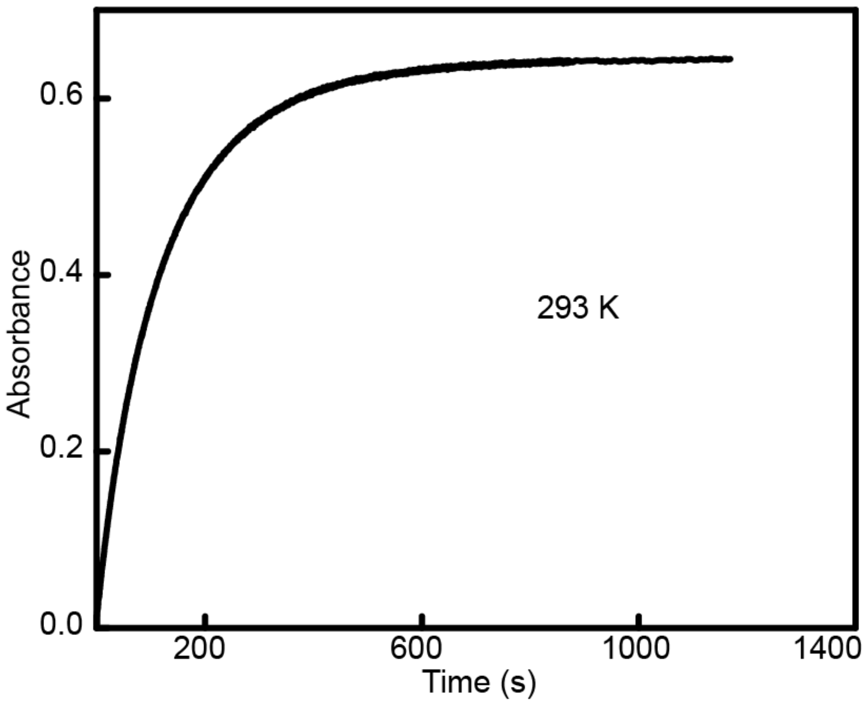

Figure S24. Plot absorbance at 780 nm vs. time in the presence of 110 μM catalyst, 0.4975 mM O_2, 45 mM of HCl and 10 mM of decamethylferrocene at 293 K. N(afa^{Cy})$_3$Fe(O)OTf was used as catalyst.

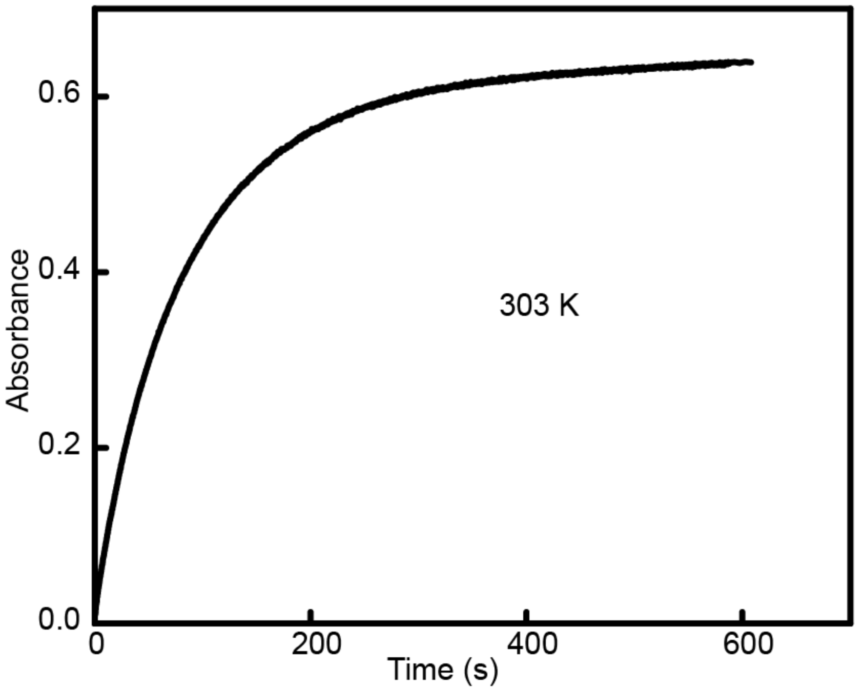

Figure S25. Plot absorbance at 780 nm vs. time in the presence of 110 μM catalyst, 0.4975 mM O_2, 45 mM of HCl and 10 mM of decamethylferrocene at 303 K. N(afa^{Cy})$_3$Fe(O)OTf was used as catalyst.

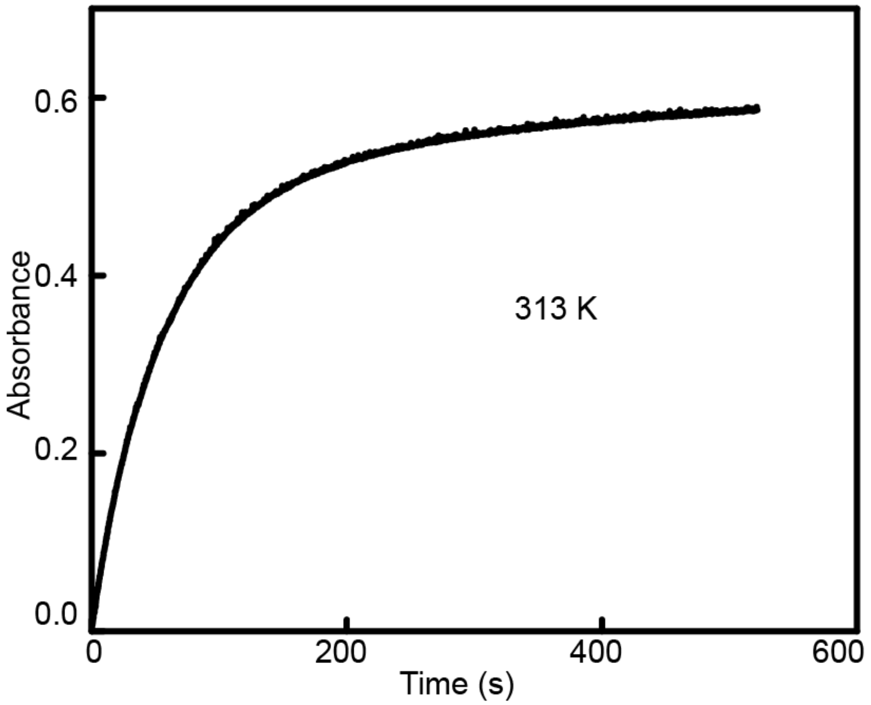

Figure S26. Plot absorbance at 780 nm vs. time in the presence of 110 μM catalyst, 0.4975 mM O_2, 45 mM of HCl and 10 mM of decamethylferrocene at 313 K. N(afa^{Cy})$_3$Fe(O)OTf was used as catalyst. Black solid line: experimental data.

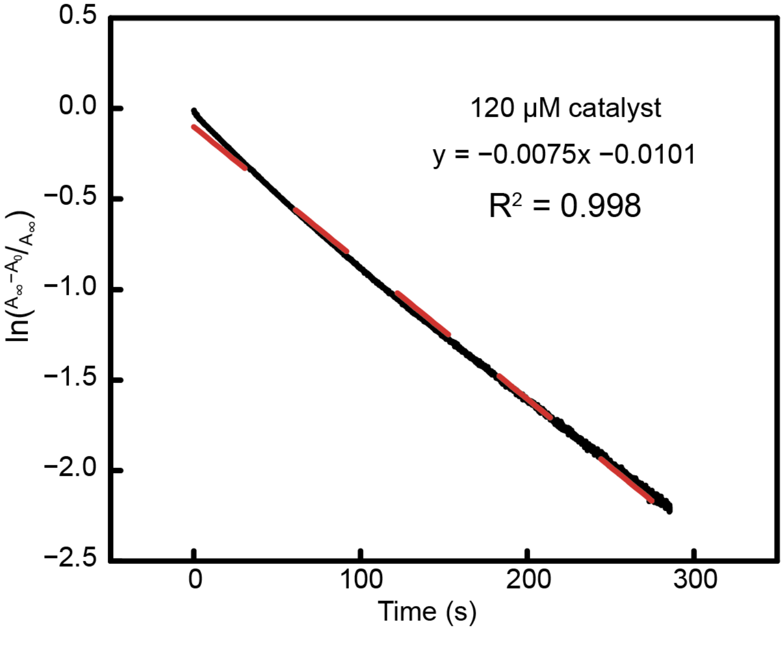

Figure S27. Plot ln(A∞−A)/A∞ vs. time (s) absorbance at 780 nm vs. time in the presence of 120 μM of catalyst, 0.4875 mM O_2, 45 mM of HCl and 10 mM of decamethylferrocene. N(afa^{Cy})$_3$Fe(O)OTf was used as catalyst. Black solid line: experimental data. Red dash line: linear fitting.

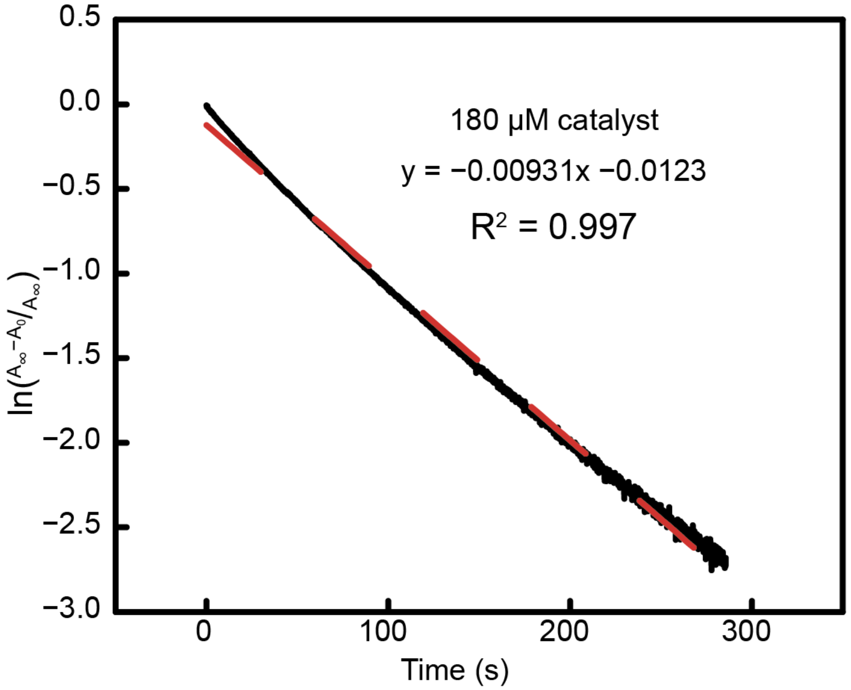

Figure S28. Plot ln(A∞−A)/A∞ vs. time (s) absorbance at 780 nm vs. time in the presence of 180 μM of catalyst, 0.4875 mM O_2, 45 mM of HCl and 10 mM of decamethylferrocene. N(afa^{Cy})$_3$Fe(O)OTf was used as catalyst. Black solid line: experimental data. Red dash line: linear fitting.

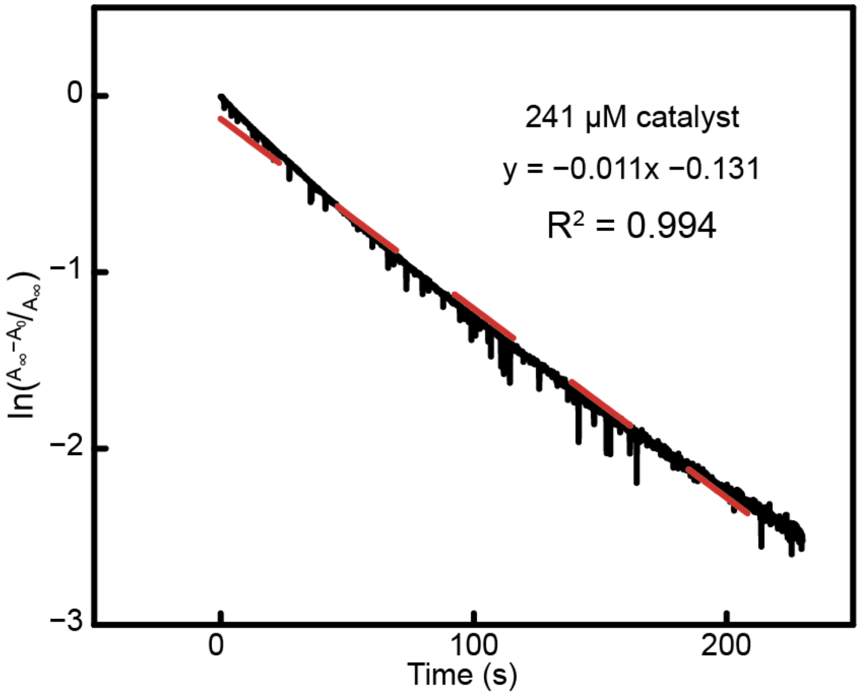

Figure S29. Plot ln(A∞−A)/A∞ vs. time (s) absorbance at 780 nm vs. time in the presence of 241 μM of catalyst, 0.4875 mM O_2, 45 mM of HCl and 10 mM of decamethylferrocene. N(afa^{Cy})$_3$Fe(O)OTf was used as catalyst. Black solid line: experimental data. Red dash line: linear fitting.

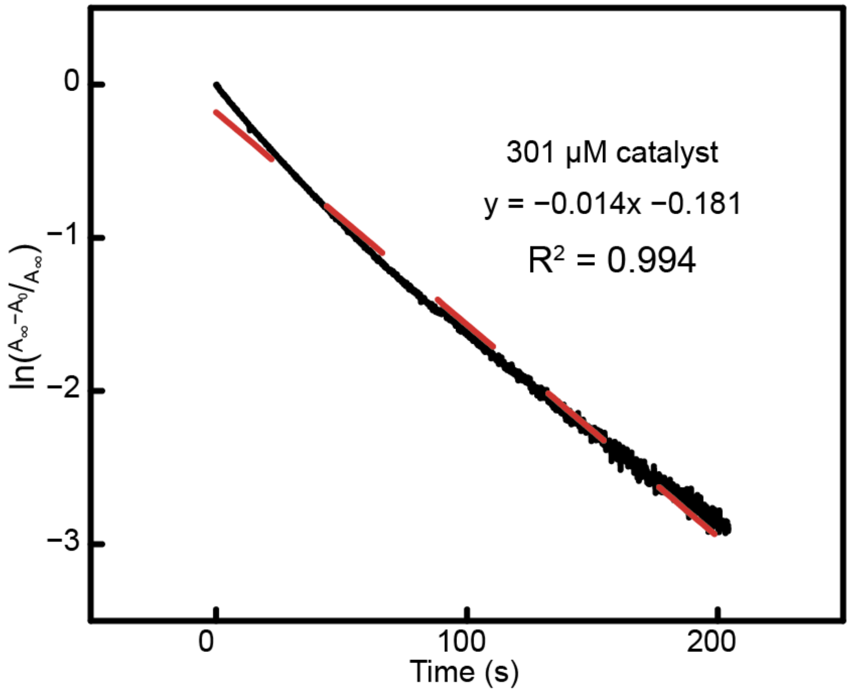

Figure S30. Plot ln(A∞−A)/A∞ vs. time (s) absorbance at 780 nm vs. time in the presence of 301 μM of catalyst, 0.4875 mM O_2, 45 mM of HCl and 10 mM of decamethylferrocene. N(afa^{Cy})$_3$Fe(O)OTf was used as catalyst. Black solid line: experimental data. Red dash line: linear fitting.

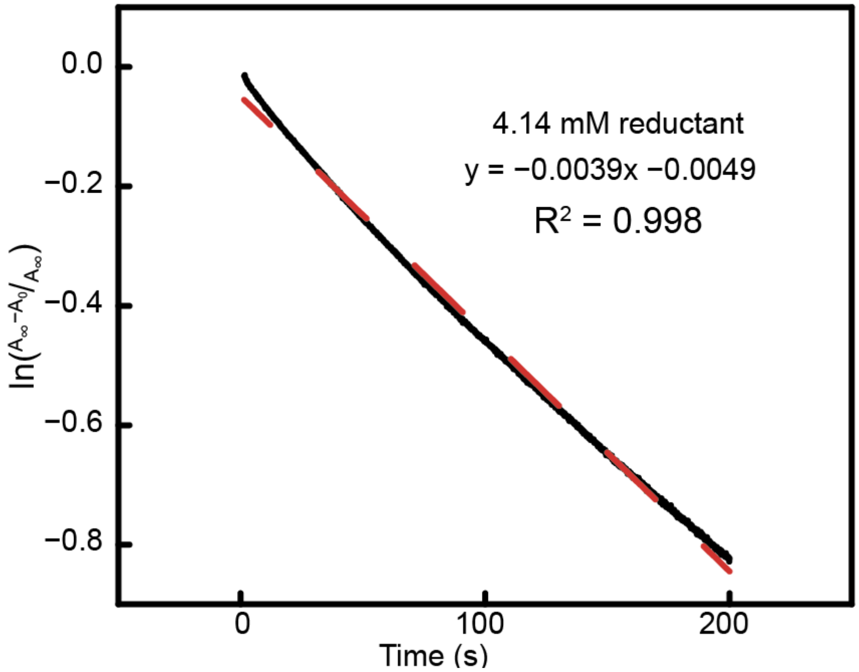

Figure S31. Plot ln(A∞−A)/A∞ vs. time (s) absorbance at 780 nm vs. time in the presence of 110 μM of catalyst, 0.4875 mM O_2, 45 mM of HCl and 4.14 mM of decamethylferrocene. N(afa^{Cy})$_3$Fe(O)OTf was used as catalyst. Black solid line: experimental data. Red dash line: linear fitting.

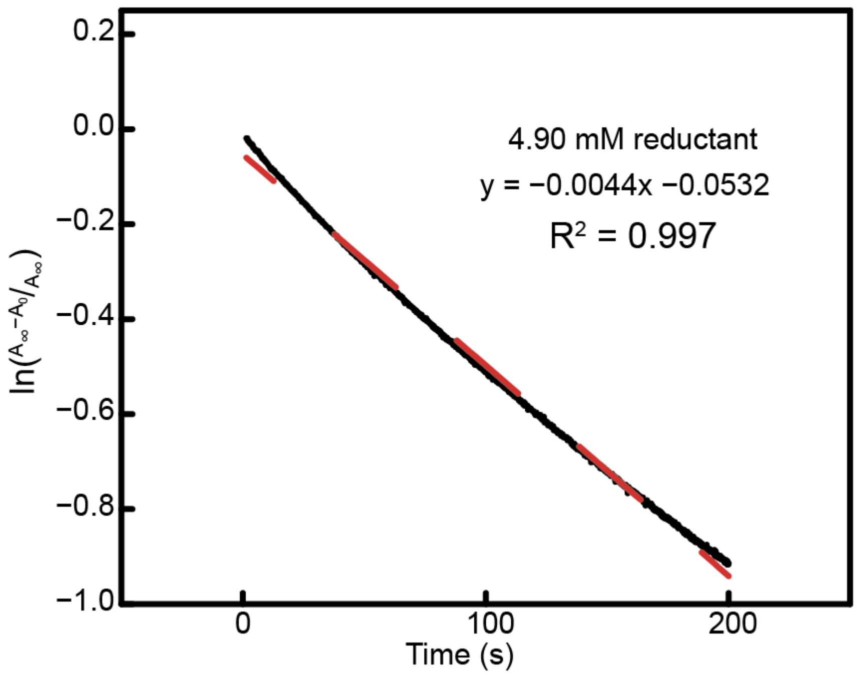

Figure S32. Plot ln(A∞−A)/A∞ vs. time (s) absorbance at 780 nm vs. time in the presence of 110 μM of catalyst, 0.4875 mM O_2, 45 mM of HCl and 4.90 mM of decamethylferrocene. N(afa^{Cy})$_3$Fe(O)OTf was used as catalyst. Black solid line: experimental data. Red dash line: linear fitting.

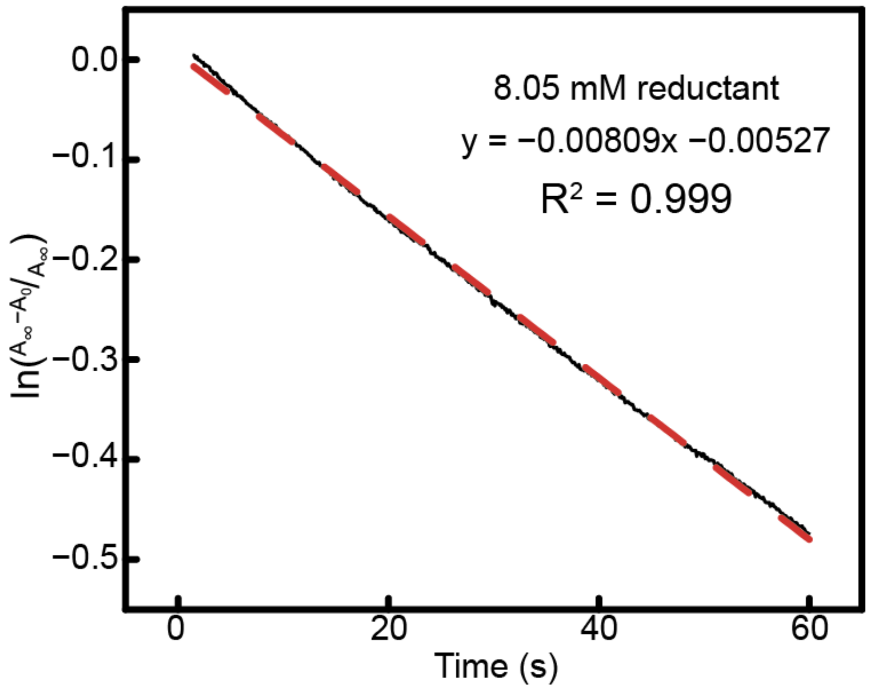

Figure S33. Plot ln(A∞−A)/A∞ vs. time (s) absorbance at 780 nm vs. time in the presence of 110 μM of catalyst, 0.4875 mM O_2, 45 mM of HCl and 8.05 mM of decamethylferrocene. N(afa^{Cy})$_3$Fe(O)OTf was used as catalyst. Black solid line: experimental data. Red dash line: linear fitting.

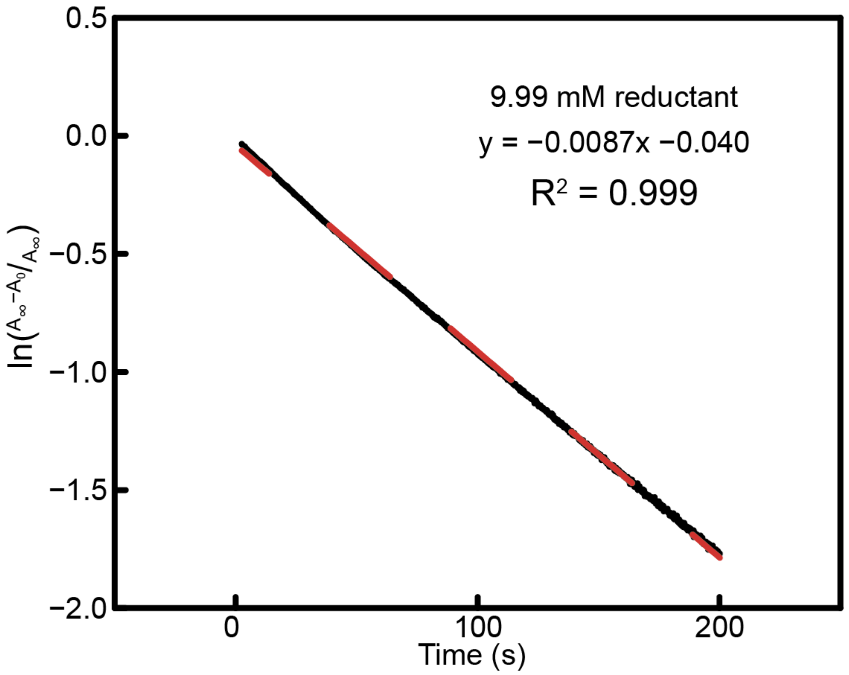

Figure S34. Plot ln(A∞−A)/A∞ vs. time (s) absorbance at 780 nm vs. time in the presence of 110 μM of catalyst, 0.4875 mM O_2, 45 mM of HCl and 9.99 mM of decamethylferrocene. $N(afa^{Cy})_3Fe(O)OTf$ was used as catalyst. Black solid line: experimental data. Red dash line: linear fitting.

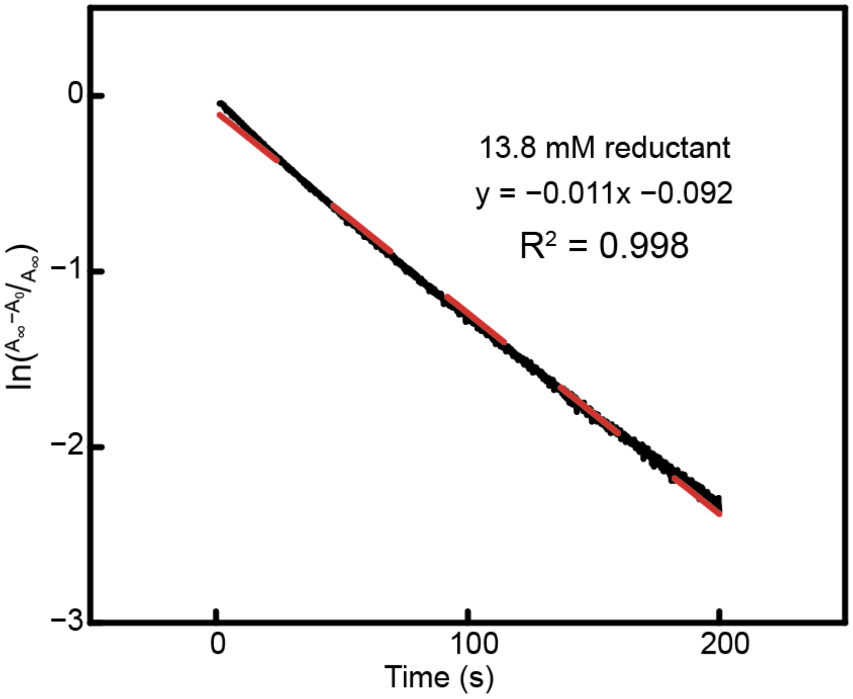

Figure S35. Plot ln(A∞−A)/A∞ vs. time (s) absorbance at 780 nm vs. time in the presence of 110 μM of catalyst, 0.4875 mM O_2, 45 mM of HCl and 13.8 mM of decamethylferrocene. $N(afa^{Cy})_3Fe(O)OTf$ was used as catalyst. Black solid line: experimental data. Red dash line: linear fitting.

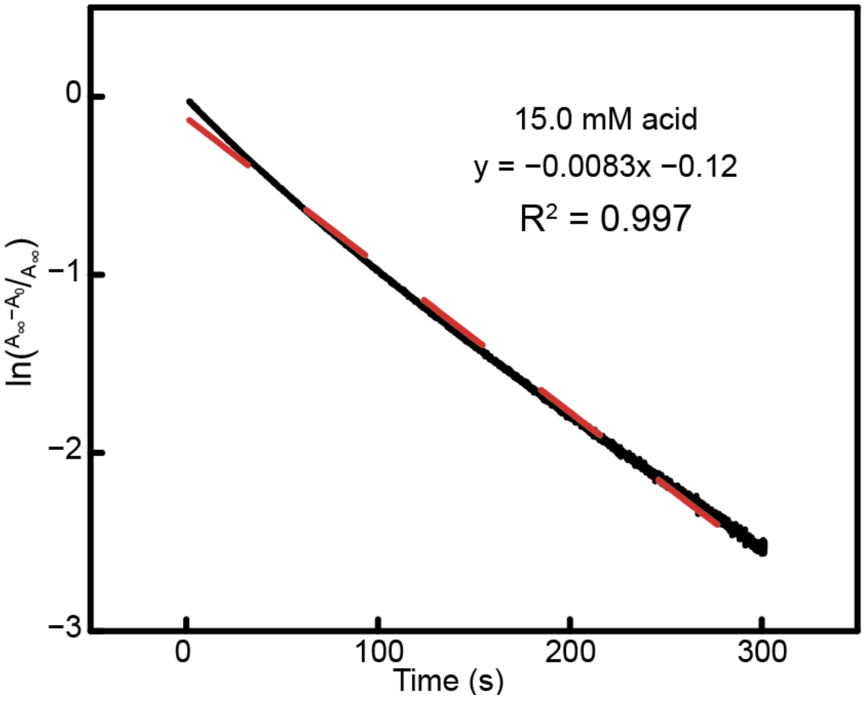

Figure S36. Plot ln(A∞−A)/A∞ vs. time (s) absorbance at 780 nm vs. time in the presence of 108 μM of catalyst, 0.4875 mM O_2, 15 mM of HCl and 10 mM of decamethylferrocene. N(afa^{Cy})$_3$Fe(O)OTf was used as catalyst. Black solid line: experimental data. Red dash line: linear fitting.

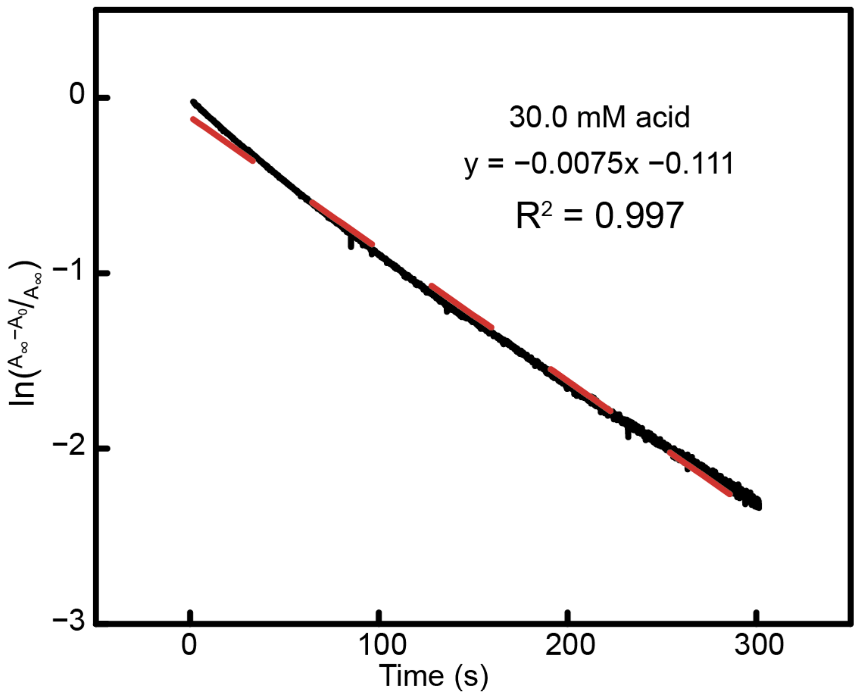

Figure S37. ln(A∞−A)/A∞ vs. time (s) absorbance at 780 nm vs. time in the presence of 108 μM of catalyst, 0.4875 mM O_2, 30 mM of HCl and 10 mM of decamethylferrocene. N(afa^{Cy})$_3$Fe(O)OTf was used as catalyst. Black solid line: experimental data. Red dash line: linear fitting.

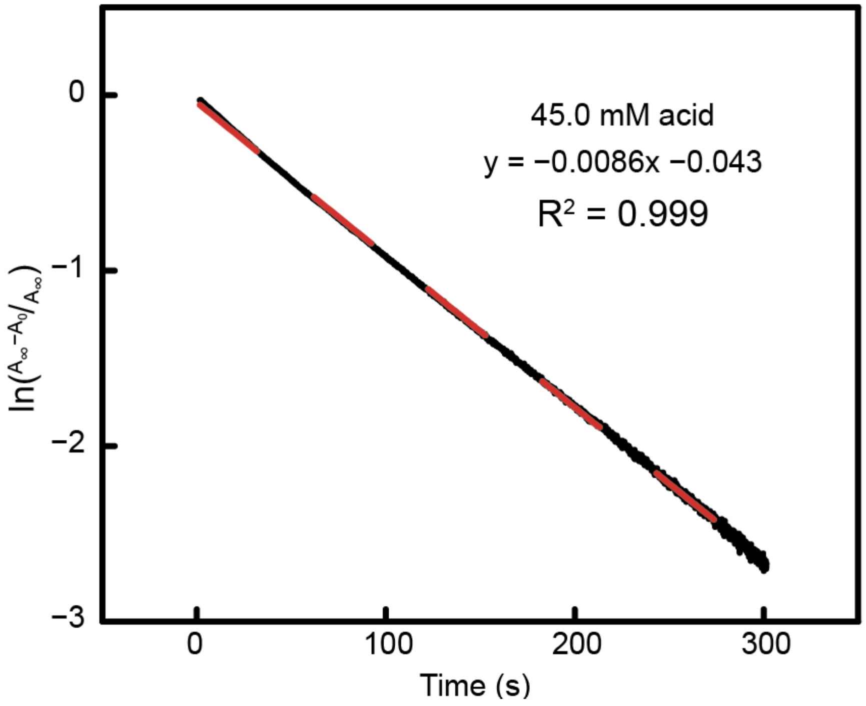

Figure S38. ln(A∞−A)/A∞ vs. time (s) absorbance at 780 nm vs. time in the presence of 108 µM of catalyst, 0.4875 mM O_2, 45 mM of HCl and 10 mM of decamethylferrocene. $N(afa^{Cy})_3Fe(O)OTf$ was used as catalyst. Black solid line: experimental data. Red dash line: linear fitting.

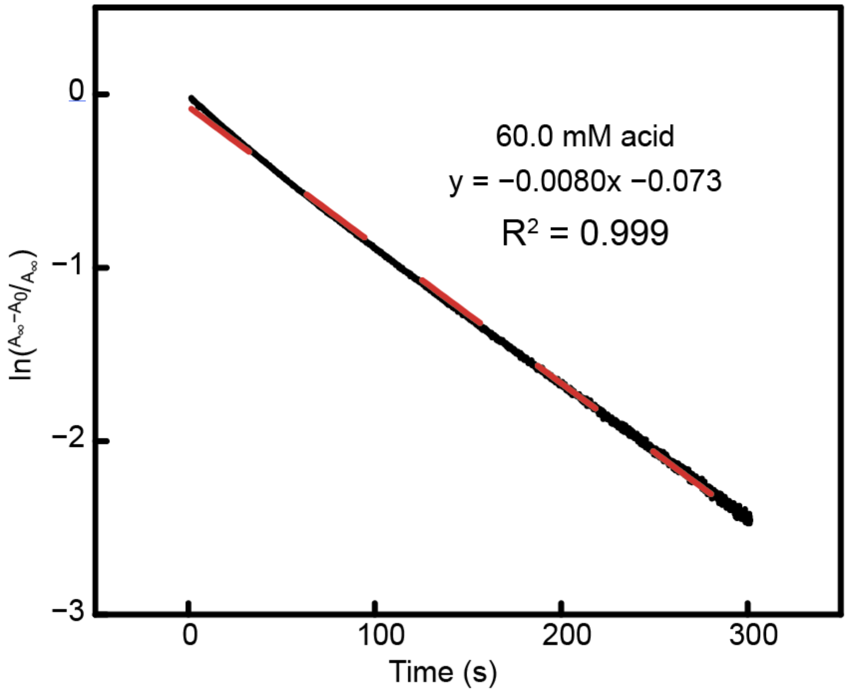

Figure S39. ln(A∞−A)/A∞ vs. time (s) absorbance at 780 nm vs. time in the presence of 108 µM of catalyst, 0.4875 mM O_2, 60 mM of HCl and 10 mM of decamethylferrocene. $N(afa^{Cy})_3Fe(O)OTf$ was used as catalyst. Black solid line: experimental data. Red dash line: linear fitting.

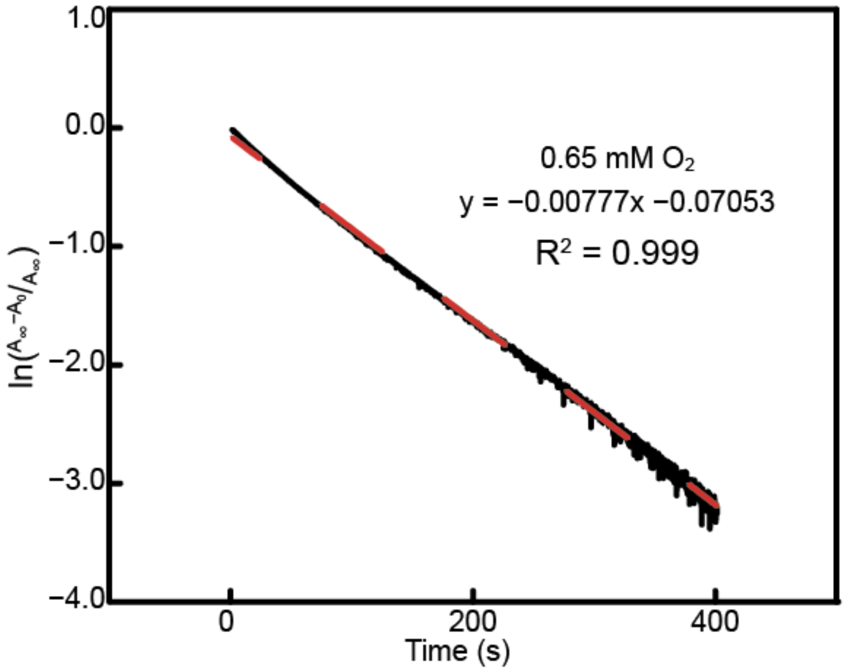

Figure S40. ln(A∞−A)/A∞ vs. time (s) absorbance at 780 nm vs. time in the presence of 110 μM of catalyst, 0.65 mM O_2, 45 mM of HCl and 10 mM of decamethylferrocene. N(afa^{Cy})$_3$Fe(O)OTf was used as catalyst. Black solid line: experimental data. Red dash line: linear fitting.

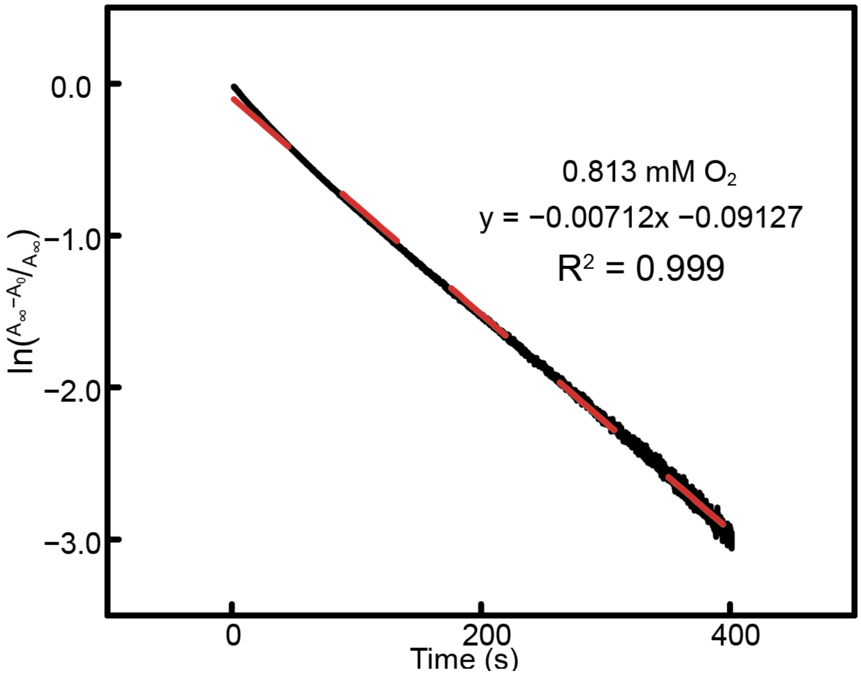

Figure S41. ln(A∞−A)/A∞ vs. time (s) absorbance at 780 nm vs. time in the presence of 110 μM of catalyst, 0.813 mM O_2, 45 mM of HCl and 10 mM of decamethylferrocene. N(afa^{Cy})$_3$Fe(O)OTf was used as catalyst. Black solid line: experimental data. Red dash line: linear fitting.

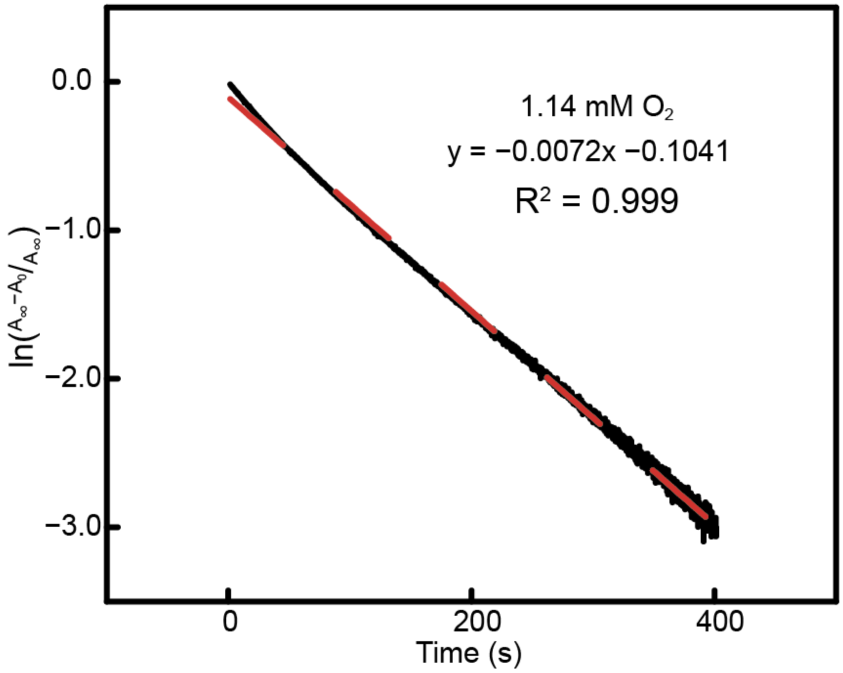

Figure S42. $\ln(A_\infty - A)/A_\infty$ vs. time (s) absorbance at 780 nm vs. time in the presence of 110 μM of catalyst, 1.14 mM O_2, 45 mM of HCl and 10 mM of decamethylferrocene. N(afa^{Cy})$_3$Fe(O)OTf was used as catalyst. Black solid line: experimental data. Red dash line: linear fitting.

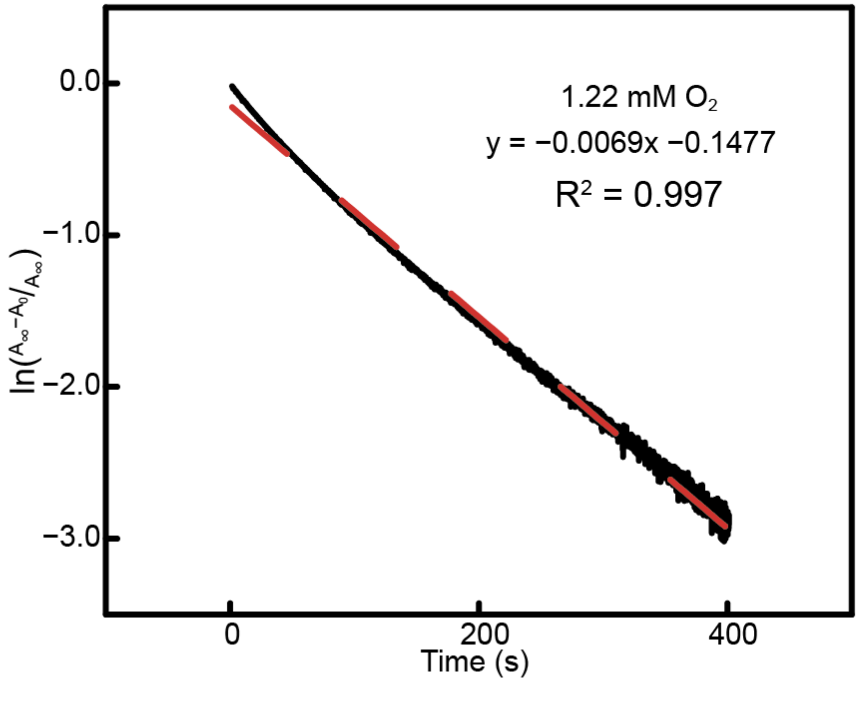

Figure S43. $\ln(A_\infty - A)/A_\infty$ vs. time (s) absorbance at 780 nm vs. time in the presence of 110 μM of catalyst, 1.22 mM O_2, 45 mM of HCl and 10 mM of decamethylferrocene. N(afa^{Cy})$_3$Fe(O)OTf was used as catalyst. Black solid line: experimental data. Red dash line: linear fitting.

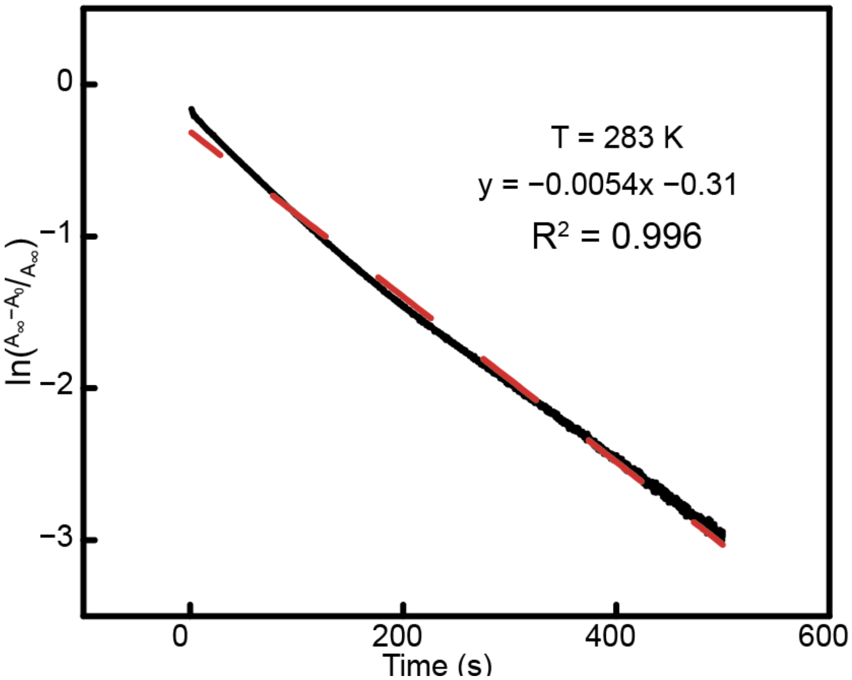

Figure S44. ln(A∞−A)/A∞ vs. time (s) absorbance at 780 nm vs. time in the presence of 110 µM of catalyst, 0.4875 mM O_2, 45 mM of HCl and 10 mM of decamethylferrocene at 283 K. N(afa^{Cy})$_3$Fe(O)OTf was used as catalyst. Black solid line: experimental data. Red dash line: linear fitting.

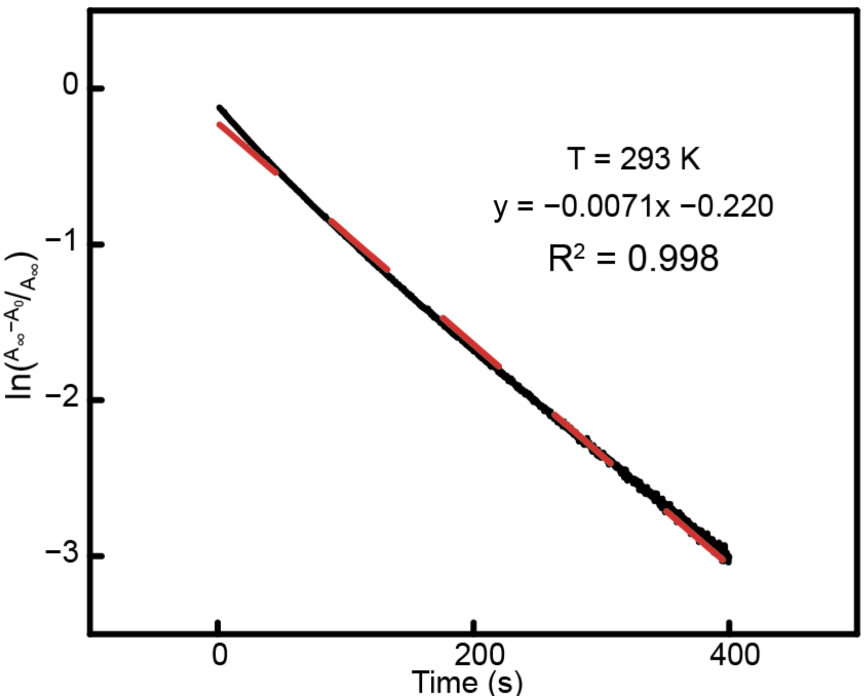

Figure S45. ln(A∞−A)/A∞ vs. time (s) absorbance at 780 nm vs. time in the presence of 110 µM of catalyst, 0.4875 mM O_2, 45 mM of HCl and 10 mM of decamethylferrocene at 293 K. N(afa^{Cy})$_3$Fe(O)OTf was used as catalyst. Black solid line: experimental data. Red dash line: linear fitting.

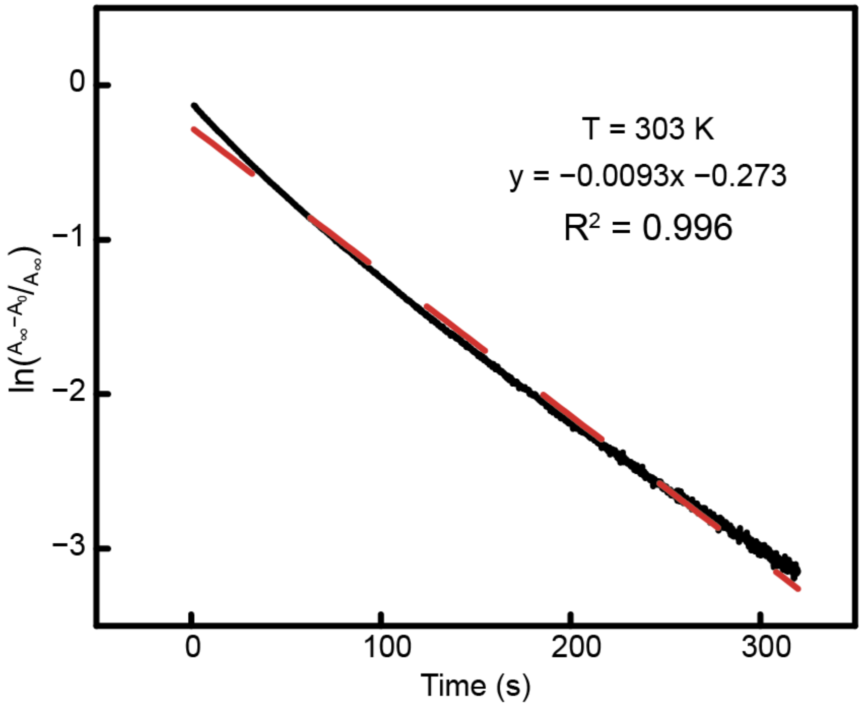

Figure S46. ln(A∞−A)/A∞ vs. time (s) absorbance at 780 nm vs. time in the presence of 110 µM of catalyst, 0.4875 mM O_2, 45 mM of HCl and 10 mM of decamethylferrocene at 303 K. N(afa^{Cy})$_3$Fe(O)OTf was used as catalyst. Black solid line: experimental data. Red dash line: linear fitting.

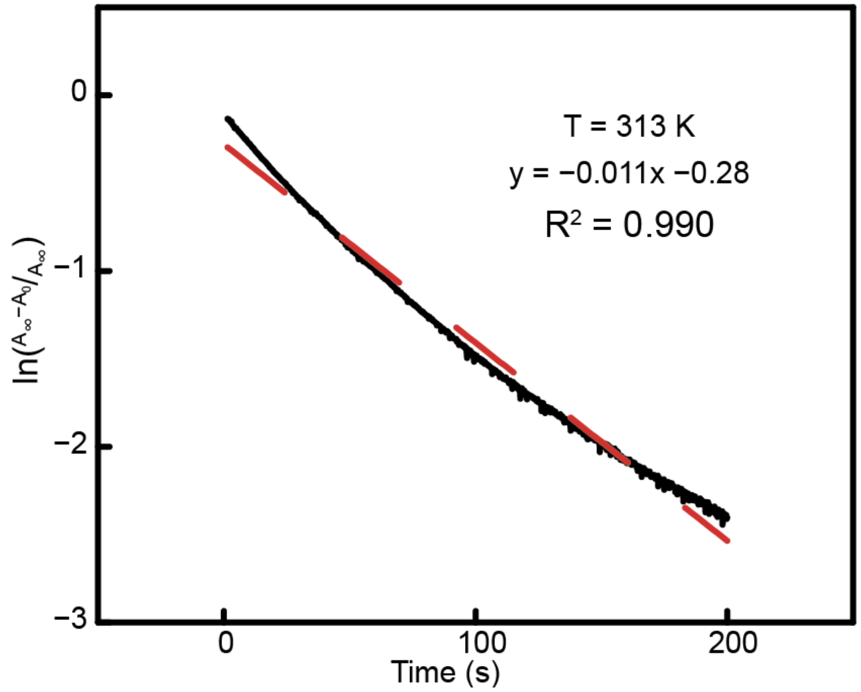

Figure S47. ln(A∞−A)/A∞ vs. time (s) absorbance at 780 nm vs. time in the presence of 110 µM of catalyst, 0.4875 mM O_2, 45 mM of HCl and 10 mM of decamethylferrocene at 313 K. N(afa^{Cy})$_3$Fe(O)OTf was used as catalyst. Black solid line: experimental data. Red dash line: linear fitting.

Calculation of kinetic parameters.

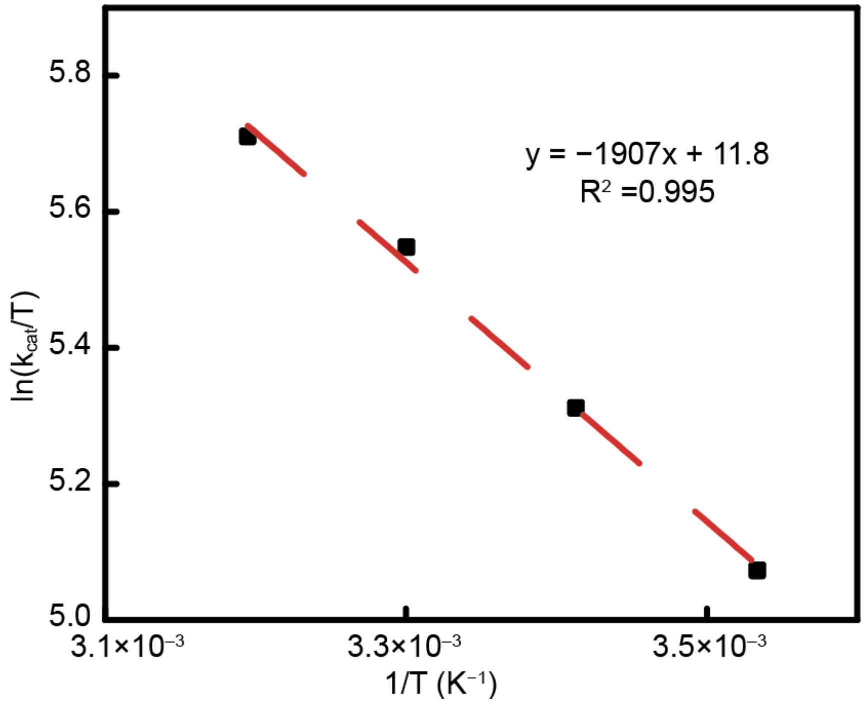

Figure S48. Eyring plot using complex **3** as ORR catalyst under various temperatures. Concentrations: [3] = 110 mM, [O2] = 0.4875 mM, [Fc*] = 10 mM, [HCl] = 45 mM.

The activation parameters were determined using the Eyring plot. Entropy ($\Delta S^{\ddagger}$) and enthalpy ($\Delta H^{\ddagger}$) were calculated from the slope and y-intercept, respectively. The Gibbs free energy of activation ($\Delta G^{\ddagger}$) was derived using the Gibbs free energy equation.

$$\ln\left(\frac{k}{T}\right) = \frac{-\Delta H^{\ddagger}}{R}\left(\frac{1}{T}\right) + \ln\left(\frac{k_B}{h}\right) + \frac{\Delta S^{\ddagger}}{R}$$

$$\frac{\Delta S^{\ddagger}}{R} = 11.82 - \ln\left(\frac{k_B}{h}\right) = -11.94$$

$$\Delta S^{\ddagger} = -23.73\ cal \cdot K^{-1} \cdot mol^{-1}$$

$$\Delta H^{\ddagger}(298\ K) = 3.79\ kcal \cdot mol^{-1}$$

$$\Delta G^{\ddagger} = \Delta H^{\ddagger} - T\Delta S^{\ddagger}$$

$$\Delta G^{\ddagger}(298\ K) = 10.86\ \mathrm{k}cal \cdot mol^{-1}$$

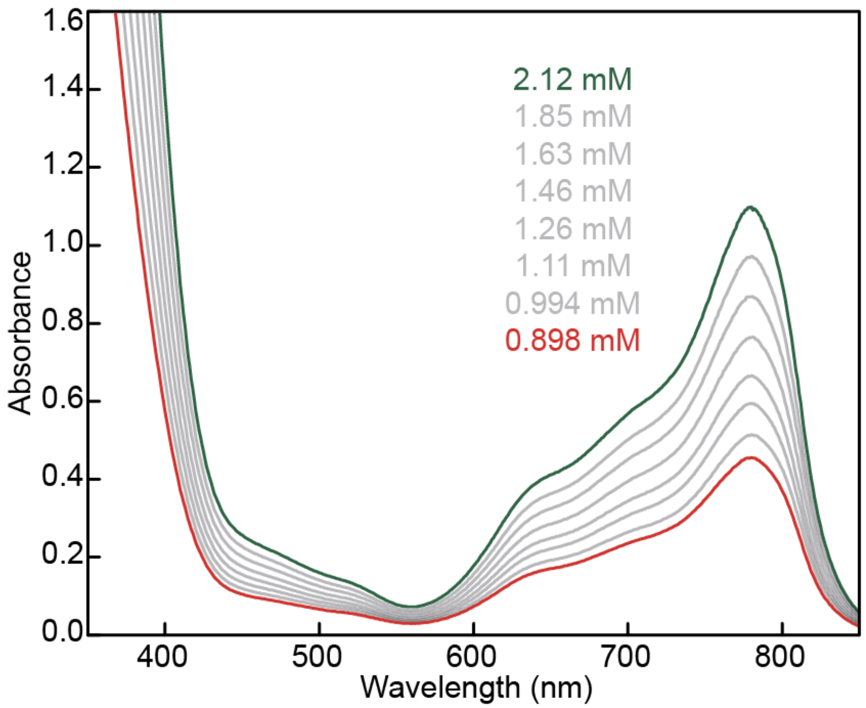

Figure S49. UV-Vis spectra of decamethylferrocenium chloride used for calibration.

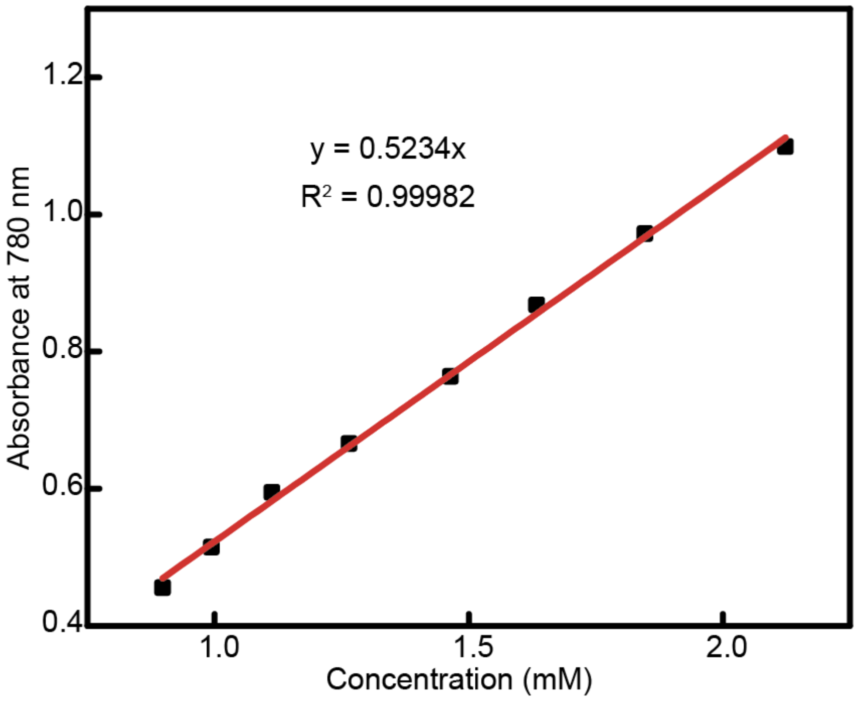

Figure S50. Calibration curve for decamethylferrocenium chloride at 780 nm.

Titration experiments for H_2O_2 determination.

Iodometric titration.

The modified method for iodometric titrations was based on a method reported by Rosenthal.[7] Following the chemical reduction experiment limiting by Fc*, 0.050 mL of the reaction from cuvette was added to a 2.05 mL of KI-saturated benzonitrile solution. Upon addition, color was changed immediately to light yellow and the UV-Vis spectra was recorded. This solution was then heated at 150 °C for 3 minutes and cooled to room temperature before the other UV-Vis spectra were collected. The amount of triiodide (λ = 365 nm, ε = 28 mM^{-1} cm^{-1}) was determined, by absorbance spectroscopy and was then correlated to the amount of H_2O_2 in the solution based on Eq.1 and Eq.2.

$$H_2O_2 + 2\,H^+ + 2\,I^- \longrightarrow I_2 + 2\,H_2O \qquad \text{(Eq. 1)}$$

$$I_2 + 2\,I^- \longrightarrow I_3^- \qquad \text{(Eq. 2)}$$

$$2\,Fc^{*} + 3\,I^- \longrightarrow 2\,Fc^{*} + I_3^- \qquad \text{(Eq. 3)}$$

Knowing that Fc^{*+} can react with iodide to regenerate Fc* and triiodide (Eq.3),[8] the heat was to make sure both Fc^{*+} and H_2O_2 were reacted to iodide completely. Accordingly, when performing iodometric titration experiments, I_3^- can be produced from either H_2O_2 or Fc^{*+}. To accurately determine the amount of I_3^- generated by H_2O_2, the Fc^{*+} from ORR was calculated by calibration curve, which aids in determining the I_3^- produced from Fc^{*+}. The remaining unaccounted I_3^- was concluded to be formed by reaction with H_2O_2 (94%).

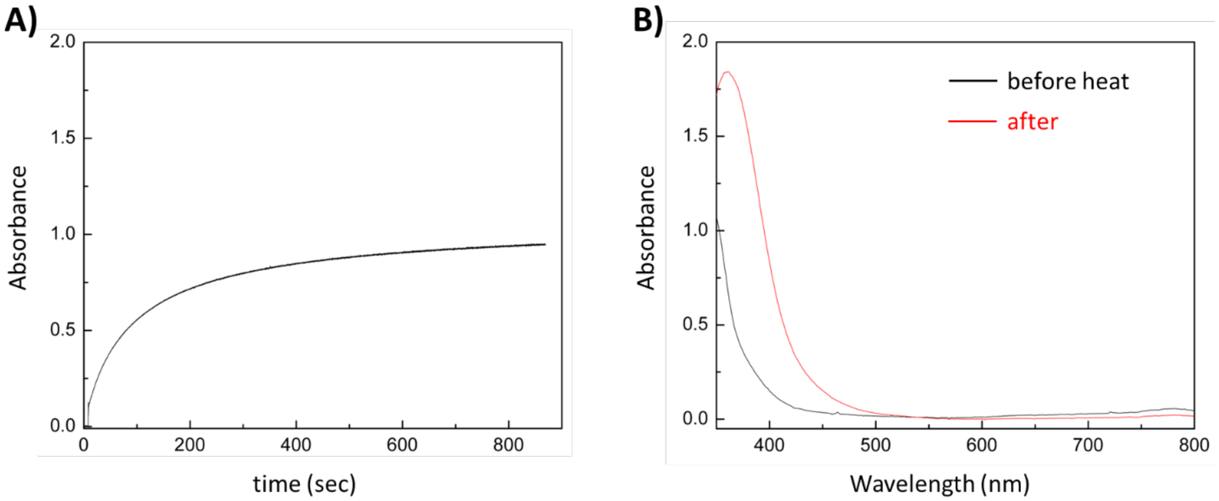

Figure S51. (**A**) Tracking absorbance at 780 nm vs. time under limiting-Fc* condition. (**B**) UV-Vis spectra for determination of the H_2O_2 by iodometric titration. Black and red traces are before and after heating at 150 °C for 3 minutes.

$$[H_2O_2]\ (mM) = \frac{Absorbance\ of\ [\mathrm{I_3}^-]_{H_2O_2}}{28}$$

$$Absorbance\ of\ [\mathrm{I_3}^-]_{H_2O_2} = Absorbance\ of\ [\mathrm{I_3}^-]_{\mathrm{total}} - Absorbance\ of\ [\mathrm{I_3}^-]_{Fc^{*+}}$$

$$Absorbance\ of\ [\mathrm{I_3}^-]_{Fc^{*+}} = 28\ \times [\mathrm{I_3}^-]_{Fc^{*+}}$$

$$[\mathrm{I_3}^-]_{Fc^{*+}}\ (mM) = \frac{1}{2}\ [Fc^{*+}] = \frac{1}{2} \times \frac{0.05}{2.1} (\frac{Abs_{Fc^{*+}} + 0.002}{0.5247})$$

Ti(O)SO$_4$ titration.

The concentration of produced H_2O_2 was determined by Ti(O)SO$_4$ as previously reported.[9-11]

Preparation of Ti(O)SO$_4$. Ti(O)SO$_4$ (0.143 g, 0.9 mmol) was added to a 2.0 M solution of sulfuric acid (9.0 mL). The Ti(O)SO$_4$ solution was heated to 50 °C to completely dissolve all the solid.

A calibration curve was obtained through a serial dilution of a stock solution of urea·H_2O_2 in PhCN:DMF 1:3 mixture while monitoring the absorbance at 408 nm. For each UV-vis sample, 1.0 mL of the stock solution was added to a mixture of 2.0 mL of DI water and 1.0 mL of ethyl acetate. After extraction, 2.0 mL of aqueous layer was transferred into a cuvette. An initial UV-vis spectrum was collected to ensure no background absorbance occurred, then 0.1 mL of the Ti(O)SO$_4$ solution was added.

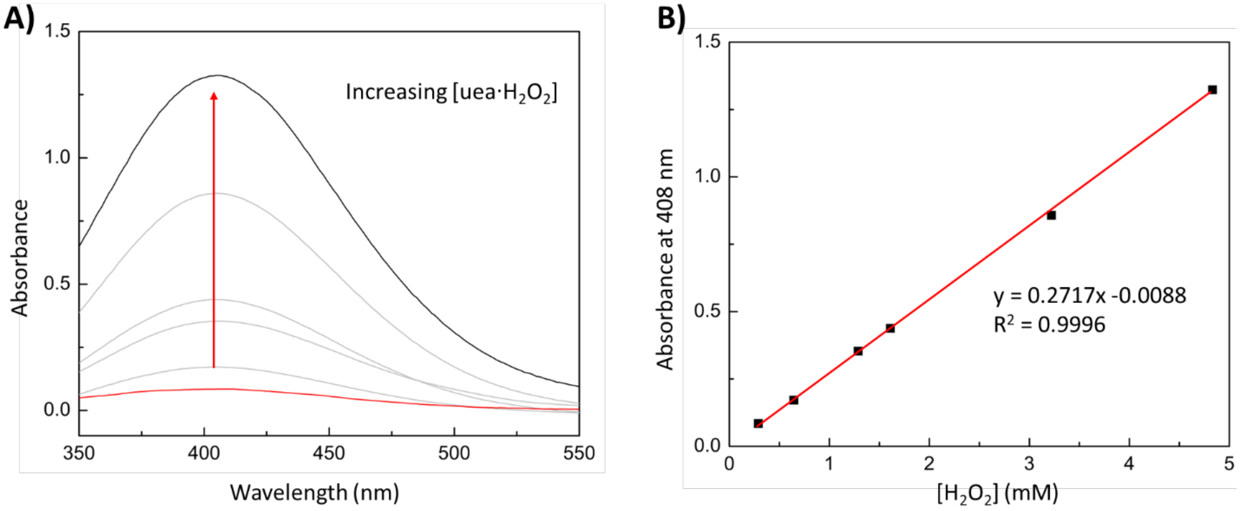

Figure S52. Calibration curve of H_2O_2 quantification using a Ti(O)SO$_4$ titration. (**A**) Serial dilution of urea•H_2O_2 in 1/3 PhCN/DMF using the above method. (**B**) Calibration made from a series of dilution of urea•H_2O_2 in 1/3 PhCN/DMF.

Following the same procedure as making calibration curve, 1.0 mL solution was taken from the chemical reduction experiments. After being extracted with 2.0 mL DI water and 1.0 mL of ethyl acetate, UV-Vis spectrum of the aqueous layer (2.0 mL) was measured before and after the addition of 0.1 mL Ti(O)SO$_4$.

However, the solution is slightly cloudy after extraction, which is possibly due to ethyl acetate droplets in the aqueous layer, which makes background not zero. The absorbance after addition decreases and further indicates that the solution turned clearer. To address the cloudiness issues and make reasonable comparison, we calibrated the whole after-spectrum and background spectrum to the decamethylferrocenium absorbance at 700 nm, as it should not change. By comparing the absorbance at 408 nm after this adjustment, the formation of H_2O_2 (79%) was confirmed.

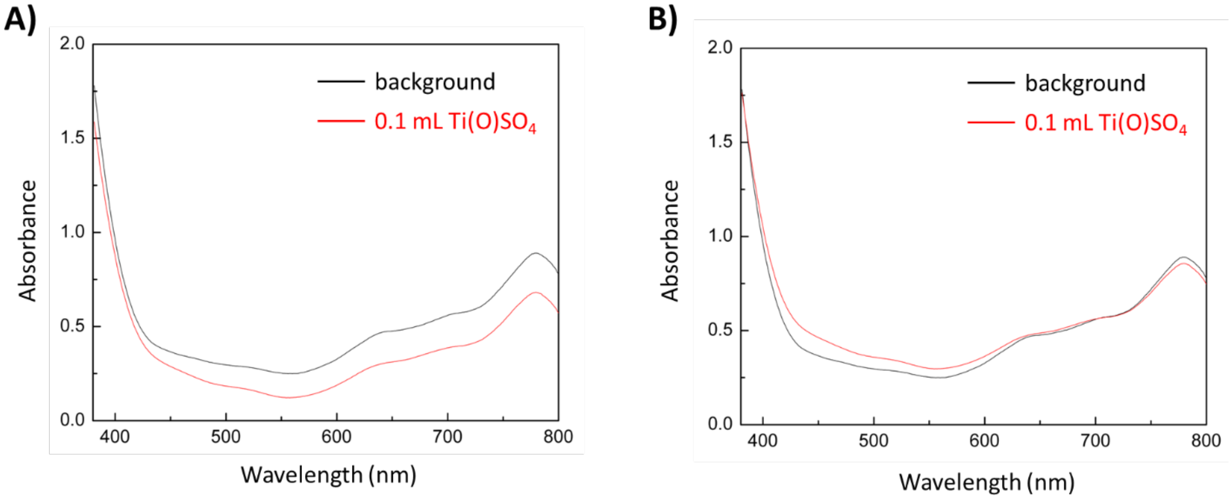

Figure S53. UV-Vis spectra of H_2O_2 titration via $Ti(O)SO_4$ method after the chemical reduction experiments (0.48 mM O_2, 45 mM HCl, 270 μM complex **3**, 10 mM Fc*). Black and red traces are the before and after the addition of 0.1 mL $Ti(O)SO_4$.(**A**) the original data. (**B**) Translate the red trace to the same absorbance as the black at 702 nm.

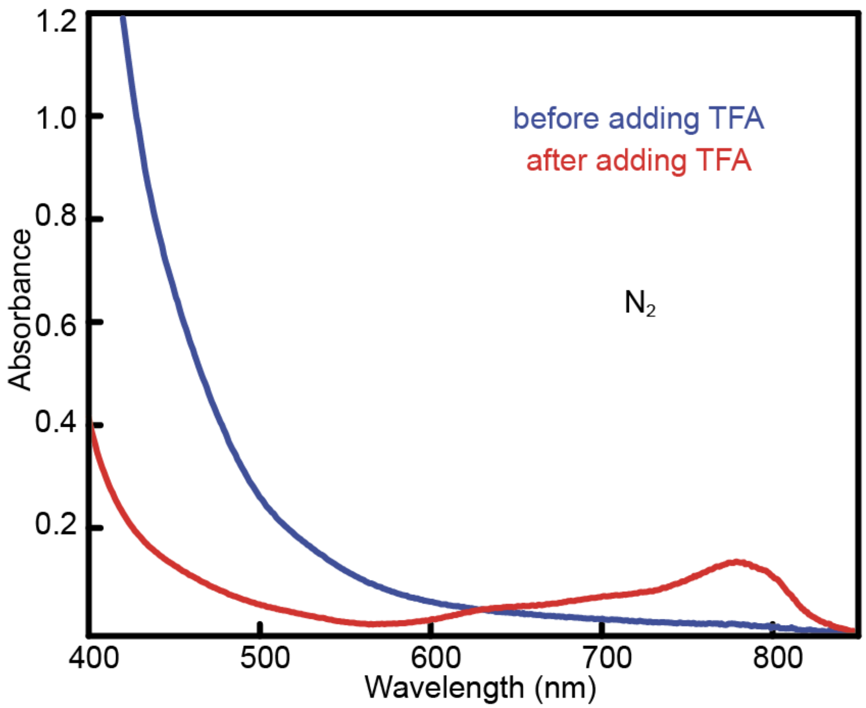

Figure S54. UV-Vis spectra N(afa^{Cy})$_3$Fe(O)OTf with decamethylferrocene in MeCN/THF mixed solution. Blue: before adding TFA. Red: after adding TFA.

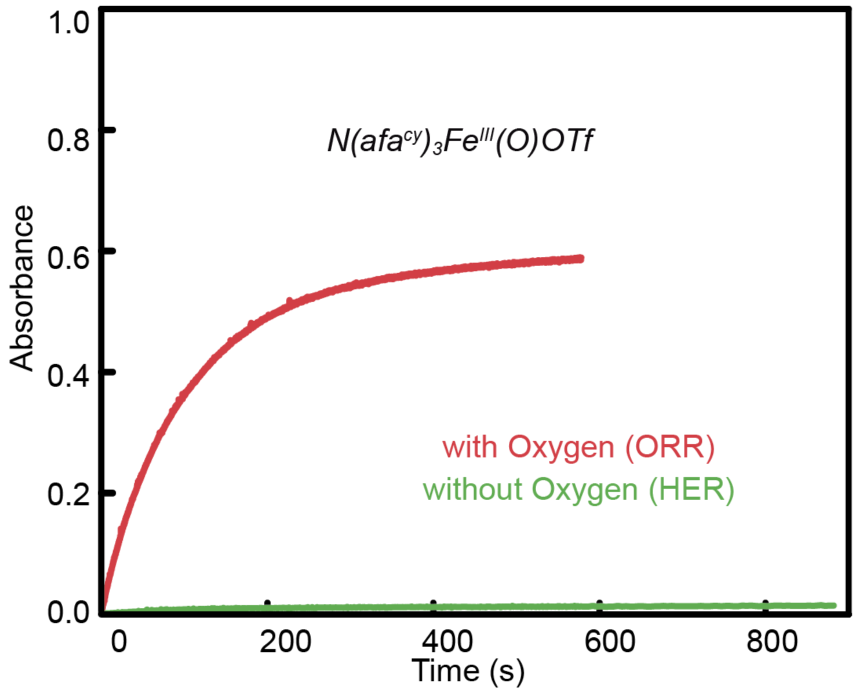

Figure S55. Plot absorbance at 780 nm vs. time in the presence of 110 μM catalyst, 45 mM of HCl and 10 mM of decamethylferrocene at 298 K for N(afa^{Cy})$_3$Fe(O)OTf. Red: with oxygen; green: without oxygen.

Electrochemistry measurements

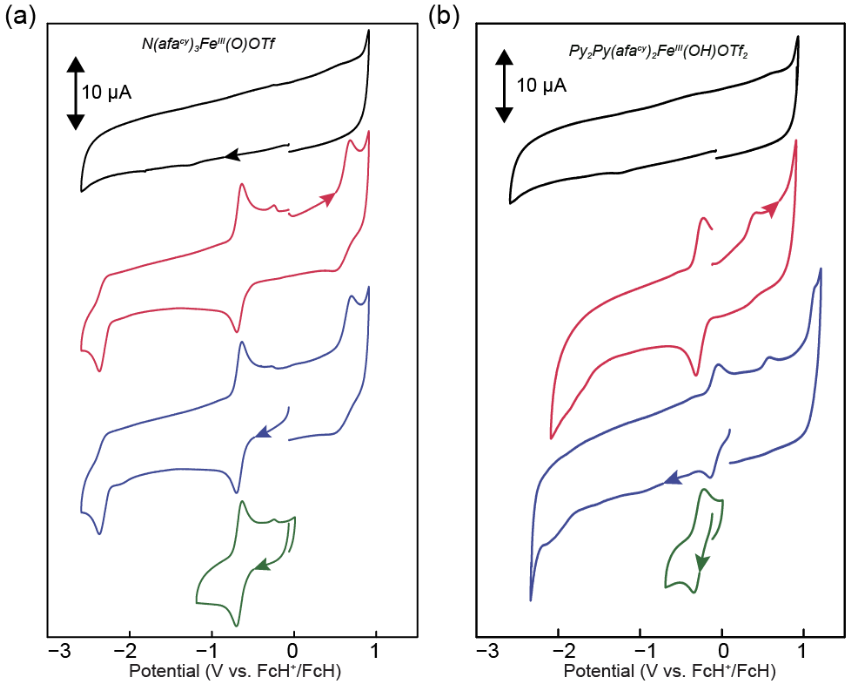

Figure S56. CVs of Fe catalysts under homogeneous conditions with no oxygen. In all cases, dry acetonitrile with 100 mM $TBAPF_6$ was used. Scan rate: 100 mV·s^{-1}, FcH$^+$/FcH was used as reference

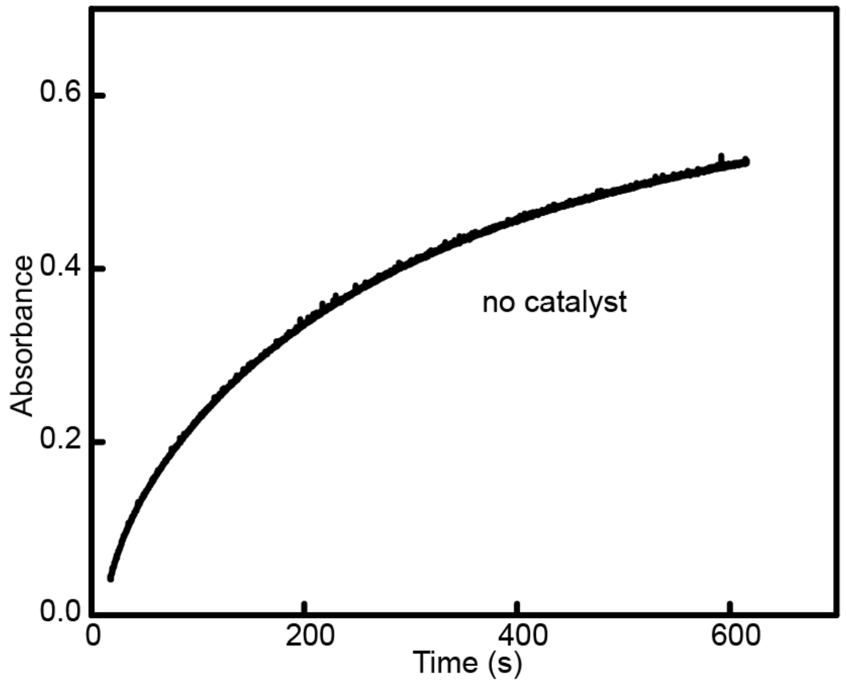

Figure S57.Plot absorbance at 780 nm vs. time in the presence of 0 catalyst, 0.4875 mM O_2, 45 mM of HCl and 10 mM of decamethylferrocene. N(afa^{Cy})$_3$Fe(O)OTf was used as catalyst.

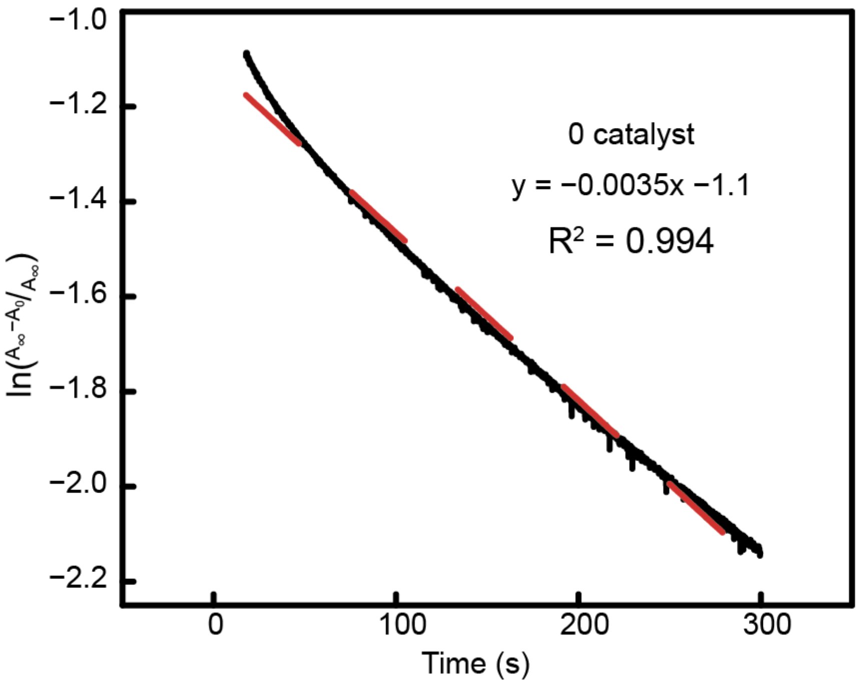

Figure S60. Plot ln(A∞−A)/A∞ vs. time (s) absorbance at 780 nm vs. time in the presence of no catalyst, 0.4875 mM O_2, 45 mM of HCl and 10 mM of decamethylferrocene. N(afa^{Cy})$_3$Fe(O)OTf was used as catalyst. Black solid line: experimental data. Red dash line: linear fitting.

Reference:

(1) Pegis, M. L.; Martin, D. J.; Wise, C. F.; Brezny, A. C.; Johnson, S. I.; Johnson, L. E.; Kumar, N.; Raugei, S.; Mayer, J. M. Mechanism of Catalytic O2 Reduction by Iron Tetraphenylporphyrin. *J. Am. Chem. Soc.* **2019**, *20*, 8315-8326.

(2) Zhang, D.; Rosch, L. E.; Crawley, M. R.; Cook, T. R. Post-synthetic modification of bis-iron(iii)-μ-oxo-porphyrin prisms to enhance oxygen reduction electrocatalysis. *Inorg. Chem. Front.* **2024**, *17*, 5557-5565.

(3) Matson, E. M.; Bertke, J. A.; Fout, A. R. Isolation of Iron(II) Aqua and Hydroxyl Complexes Featuring a Tripodal H-bond Donor and Acceptor Ligand. *Inorg. Chem.* **2014**, *9*, 4450-4458.

(4) Gordon, Z.; Miller, T. J.; Leahy, C. A.; Matson, E. M.; Burgess, M.; Drummond, M. J.; Popescu, C. V.; Smith, C. M.; Lord, R. L.; Rodríguez-López, J.; et al. Characterization of Terminal Iron(III)–Oxo and Iron(III)–Hydroxo Complexes Derived from O2 Activation. *Inorg. Chem.* **2019**, *23*, 15801-15811.

(5) Drummond, M. J.; Ford, C. L.; Gray, D. L.; Popescu, C. V.; Fout, A. R. Radical Rebound Hydroxylation Versus H-Atom Transfer in Non-Heme Iron(III)-Hydroxo Complexes: Reactivity and Structural Differentiation. *J. Am. Chem. Soc.* **2019**, *16*, 6639-6650.

(6) James, H. J.; Broman, R. F. Modified winkler determination of oxygen in dimethylformamide: oxygen solubility as a function of partial pressure. *Anal. Chim. Acta* **1969**, *2*, 411-417.
(7) Cai, Q.; Tran, L. K.; Qiu, T.; Eddy, J. W.; Pham, T.-N.; Yap, G. P. A.; Rosenthal, J. An Easily Prepared Monomeric Cobalt(II) Tetrapyrrole Complex That Efficiently Promotes the 4e–/4H+ Peractivation of O2 to Water. *Inorg. Chem.* **2022**, *14*, 5442-5451.
(8) Fukuzumi, S.; Kotani, H.; Lucas, H. R.; Doi, K.; Suenobu, T.; Peterson, R. L.; Karlin, K. D. Mononuclear Copper Complex-Catalyzed Four-Electron Reduction of Oxygen. *J. Am. Chem. Soc.* **2010**, *20*, 6874-6875.
(9) Cook, E. N.; Hooe, S. L.; Dickie, D. A.; Machan, C. W. Homogeneous Catalytic Reduction of O2 to H2O by a Terpyridine-Based FeN3O Complex. *Inorg. Chem.* **2022**, *22*, 8387-8392.
(10) Cook, E. N.; Dickie, D. A.; Machan, C. W. Catalytic Reduction of Dioxygen to Water by a Bioinspired Non-Heme Iron Complex via a 2+2 Mechanism. *J. Am. Chem. Soc.* **2021**, *40*, 16411-16418.
(11) Santra, A.; Das, A.; Kaur, S.; Jain, P.; Ingole, P. P.; Paria, S. Catalytic reduction of oxygen to water by non-heme iron complexes: exploring the effect of the secondary coordination sphere proton exchanging site. *Chem. Sci* **2024**, *11*, 4095-4105.
